# CaMKII-dependent non-canonical RIG-I pathway promotes influenza virus propagation in the acute-phase of infection

**DOI:** 10.1128/mbio.00087-24

**Published:** 2024-11-27

**Authors:** Shinichiro Hama, Miho Watanabe-Takahashi, Hiroki Nishimura, Jumpei Omi, Masakazu Tamada, Takashi Saitoh, Katsumi Maenaka, Yuta Okuda, Aoi Ikegami, Asami Kitagawa, Koudai Furuta, Kana Izumi, Eiko Shimizu, Takashi Nishizono, Makoto Fujiwara, Tomohiro Miyasaka, Shigeo Takamori, Hiroshi Takayanagi, Keizo Nishikawa, Toshihiko Kobayashi, Noriko Toyama-Sorimachi, Makoto Yamashita, Toshiya Senda, Takatsugu Hirokawa, Haruhiko Bito, Kiyotaka Nishikawa

**Affiliations:** 1 Department of Molecular Life Sciences, Graduate School of Life and Medical Sciences, Doshisha University, Kyotanabe, Japan; 2 Department of Health Chemistry, Graduate School of Pharmaceutical Sciences, The University of Tokyo, Bunkyo-ku, Tokyo, Japan; 3 Department of Medicinal Chemistry, Faculty of Pharmaceutical Sciences, Hokkaido University of Science, Sapporo, Hokkaido, Japan; 4 Laboratory of Biomolecular Science and Center for Research and Education on Drug Discovery, Faculty of Pharmaceutical Sciences, Hokkaido University, Sapporo, Hokkaido, Japan; 5 Global Station for Biosurfaces and Drug Discovery, Hokkaido University, Sapporo, Hokkaido, Japan; 6 Division of Pathogen Structure, International Institute for Zoonosis Control, Hokkaido University, Sapporo, Hokkaido, Japan; 7 Institute for Vaccine Research and Development, HU-IVReD, Hokkaido University, Sapporo, Hokkaido, Japan; 8 Department of Physiology and Anatomy, Faculty of Pharmacy, Nihon University, Funabashi, Japan; 9 Laboratory of Neural Membrane Biology, Graduate School of Brain Science, Doshisha University, Kyoto, Japan; 10 Department of Immunology, Graduate School of Medicine and Faculty of Medicine, The University of Tokyo, Bunkyo-ku, Tokyo, Japan; 11 Department of Cell Biology and Metabolic Biochemistry, Graduate School of Life and Medical Sciences, Doshisha University, Kyotanabe, Japan; 12 Division of Human Immunology, International Vaccine Design Center, The Institute of Medical Science, The University of Tokyo, Minato-ku, Tokyo, Japan; 13 Department of Clinical Infectious Diseases, Aichi Medical University, Nagakute, Japan; 14 Structural Biology Research Center, Institute of Materials Structure Science, High Energy Accelerator Research Organization (KEK), Tsukuba, Ibaraki, Japan; 15 Transborder Medical Research Center, University of Tsukuba, Tsukuba, Ibaraki, Japan; 16 Division of Biomedical Science, Institute of Medicine, University of Tsukuba, Tsukuba, Ibaraki, Japan; 17 Department of Neurochemistry, Graduate School of Medicine, The University of Tokyo, Bunkyo-ku, Tokyo, Japan; 18 International Research Center for Neurointelligence (WPI-IRCN), The University of Tokyo, Bunkyo-ku, Tokyo, Japan; Icahn School of Medicine at Mount Sinai, New York, New York, USA

**Keywords:** CaMKII, peptide library screening, influenza virus, RIG-I, cap-snatching

## Abstract

Ca^2+^/calmodulin-dependent protein kinase II (CaMKII) is one of hundreds of host-cell factors involved in the propagation of type A influenza virus (IAV), although its mechanism of action is unknown. Here, we identified CaMKII inhibitory peptide M3 by targeting its kinase domain using affinity-based screening of a tailored random peptide library. M3 inhibited IAV cytopathicity and propagation in cells by specifically inhibiting the acute-phase activation of retinoic acid-inducible gene I (RIG-I), which is uniquely regulated by CaMKII. Downstream of the RIG-I pathway activated TBK1 and then IRF3, which induced small but sufficient amounts of transcripts of the genes for IFN α/β to provide the capped 5’-ends that were used preferentially as primers to synthesize viral mRNAs by the cap-snatching mechanism. Importantly, knockout of *RIG-I* in cells almost completely inhibited the expression of IFN mRNAs and subsequent viral NP mRNA early in infection (up to 6 h after infection), which then protected cells from cytopathicity 24 h after infection. Thus, CaMKII-dependent acute-phase activation of RIG-I promoted IAV propagation, whereas the canonical RIG-I pathway stimulated antiviral activity by inducing large amounts of mRNA for IFNs and then for antiviral proteins later in infection. Co-administration of M3 with IAV infection rescued mice from the lethality and greatly reduced proinflammatory cytokine mRNA expression in the lung, indicating that M3 is highly effective against IAV *in vivo*. Thus, regulation of the CaMKII-dependent non-canonical RIG-I pathway may provide a novel host-factor-directed antiviral therapy.

**IMPORTANCE:**

The recent emergence of IAV strains resistant to commonly used therapeutic agents that target viral proteins has exacerbated the need for innovative strategies. Here, we originally identified CaMKII-inhibitory peptide M3, which efficiently inhibits IAV-lethality *in vitro* and *in vivo*. M3 specifically inhibited the acute-phase activation of RIG-I, which is a novel pathway to promote IAV propagation. Thus, this pathway acts in an opposite manner compared with the canonical RIG-I pathway, which plays essential roles in antiviral innate immune response later in infection. The CaMKII-dependent non-canonical RIG-I pathway can be a promising and novel drug target for the treatment of infections.

## INTRODUCTION

Influenza A virus (IAV) is a major causative pathogen for severe respiratory infections and causes 250,000–500,000 annual fatalities worldwide (1–3). The recent emergence of IAV strains resistant to commonly used therapeutic agents, such as neuraminidase (NA) inhibitors and viral endonuclease inhibitors, which target essential viral proteins, has accelerated the development of new therapies that target cellular proteins used for viral propagation (4, 5). Previously, genome-wide RNA interference screening identified 295 host-cell factors required for early-stage IAV replication in A549 human lung epithelial cells (6). Among them, 10 proteins, including nuclear trafficking protein CSE1L and Ca^2+^/calmodulin-dependent protein kinase IIβ(CaMKIIβ), are needed for the post-entry processes of IAV replication. Furthermore, the CaMKII inhibitor KN-93 suppressed IAV replication in cells, indicating that CaMKII is a promising target to protect against IAV infection. However, the efficacy of CaMKII inhibitors has not been shown in an animal infection model, and, importantly, the molecular function of CaMKII in IAV replication is unknown.

CaMKII plays an important role in a wide range of intracellular signaling events that are associated with diverse neuronal functions (7–13), inflammation, and cardiovascular pathology (14–18). CaMKII holoenzyme is comprised of a large symmetrical complex of 12 monomers assembled through the interaction of multiple COOH-terminal association domains (19–22) that results in the cooperative activation of each CaMKII by Ca^2+^/CaM (23). Recently, among the inhibitors of CaMKII (24, 25), the most widely used inhibitor, KN-93, has been shown to bind directly to Ca^2+^/CaM to inhibit its interaction with CaMKII (26); thus, it may have off-target effects on other Ca^2+^/CaM-dependent molecules.

Retinoic acid-inducible gene I (RIG-I), a member of the RIG-I-like receptor (RLR) family, is a key sensor of viral RNAs for the transduction of antiviral signals (27–29). Activated RIG-I interacts with mitochondrial antiviral signaling protein (MAVS) to induce its activation and oligomerization, stimulating the formation of a signal complex. The complex activates IκB kinase (IKK) and Tank binding kinase-1 (TBK1), resulting in the activation of nuclear factor-κB (NF-κB) and interferon regulatory factor (IRF) 3/7, respectively. This activation induces the expression of type I interferon (IFN) and proinflammatory cytokines, which inhibit IAV propagation through enhanced expression of a series of IFN-stimulated genes (ISGs) (30). In contrast, the induction of mRNAs for type I IFN and proinflammatory cytokines can enhance IAV propagation. This is due to cap-snatching (31), in which viral RNA-dependent RNA polymerase cleaves 5′ 7-methyl guanosine (m7G) caps from the predominant host-cell pre-mRNAs, using the caps as primers to initiate viral mRNA transcription (32, 33). However, the mechanism by which IAV propagates efficiently by cap-snatching in the presence of large amounts of antiviral host protein mRNAs induced by infection is unknown.

In this study, we identified CaMKII inhibitory peptide M3 by targeting the kinase domain using affinity-based screening of a tailored random peptide library, which is highly effective for identifying high-affinity binding motifs against multi-subunit target proteins (34–38). M3, but not KN-93, efficiently inhibited IAV propagation *in vitro* and *in vivo*. M3 specifically inhibited the CaMKII-dependent acute-phase activation of RIG-I, which provides small but sufficient amounts of the capped 5’-ends to promote viral mRNA synthesis. The discovery of this novel pathway provides a promising target for the treatment of influenza.

## RESULTS

### Multivalent peptide library screening identifies a series of CaMKII inhibitory peptides

**Fig 1:**
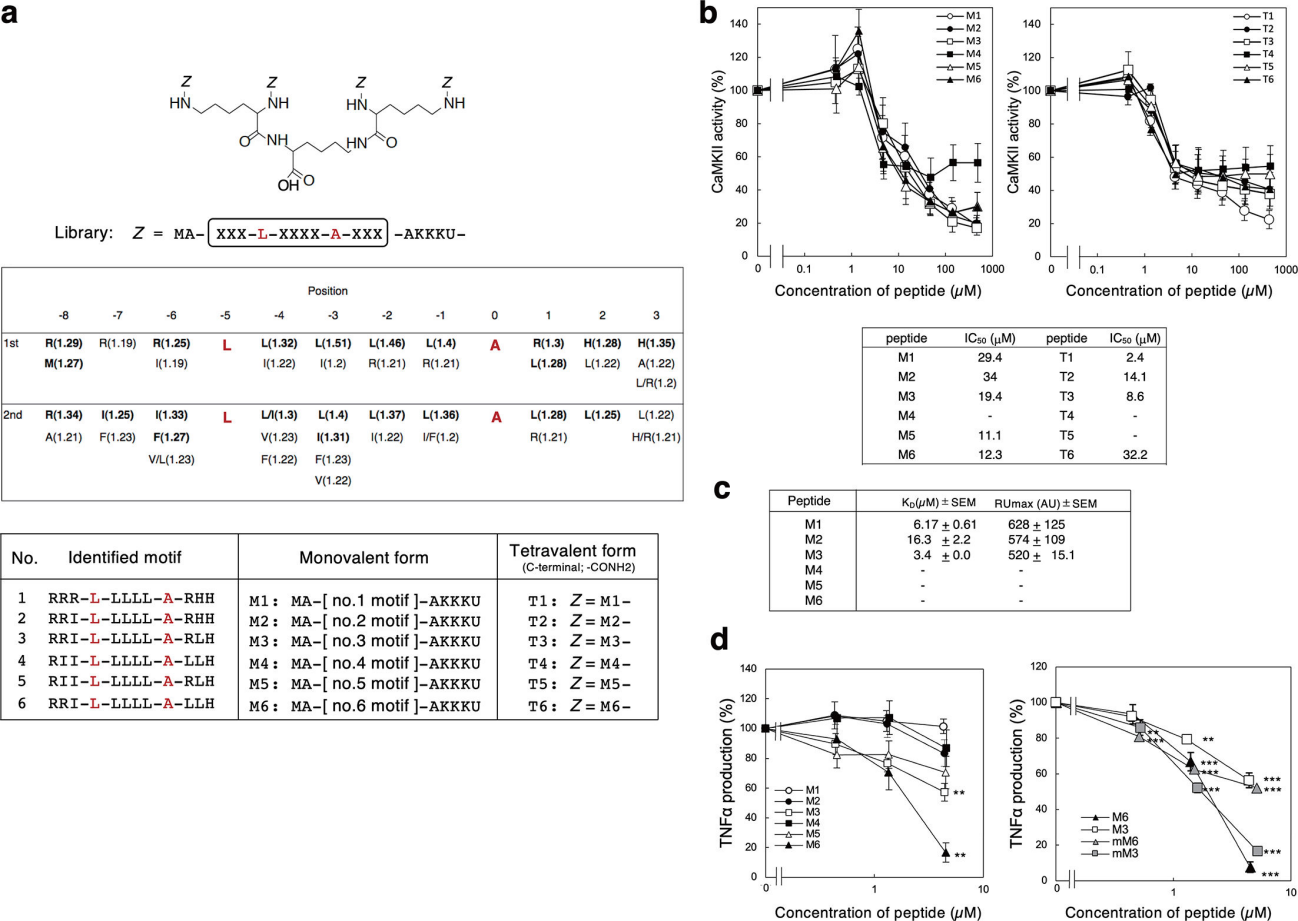
Multivalent peptide library screen identified CaMKII inhibitory peptides. (a) The tetravalent peptide screening library comprised tetravalent compounds with four randomized peptides of sequence. Met-Ala-X-X-X-Leu-X-X-X-X-Ala-X-X-X-Ala-Lys-Lys-Lys-U (U; amino hexanoic acid), where X indicates any amino acid except Cys. Ala was introduced at position 0, the phosphorylation site of substrates, to prevent the library from serving as substrates (upper panel). For compounds bound to CaMKII-KD, values in parentheses next to amino acids indicate the relative selectivity and amino acids with strong selectivity are in bold. The first and second designations (middle panel) refer to the two screens, and monovalent and tetravalent peptides with the six CaMKII-KD binding motifs were synthesized as candidate compounds (lower panel). (b) The inhibitory effects of monovalent peptides (upper left panel) or tetravalent peptides (upper right panel) on the kinase activity of CaMKII-KD with autocamtide-2 peptide as the phosphorylation substrate. Data are presented as a percentage of the control value without peptide (*n* = 3–7, mean ± SEM). IC_50_ values of peptides are shown (lower panel). (c) Kinetics of the binding of monovalent peptide to immobilized CaMKII-KD was analyzed using the BIAcore system with an arbitrary resonance unit (RU) to indicate peptide binding (*n* = 3, mean ± SEM). (d) The inhibitory effects of monovalent peptides on the production of TNFα.RAW264.7 cells were incubated with each peptide for 30 min and then treated with LPS for 24 h. TNFα production in the culture medium was determined by ELISA. Data are presented as a percentage of the control value without peptide (*n* = 3–12, mean ± SEM). **P < 0.01; ***P < 0.005 (compared with the control by ANOVA followed by one-sided Dunnett’s test).

Previously, we developed a multivalent random peptide library to identify high-affinity peptides that bind to multi-subunit proteins or those that oligomerize, such as Shiga toxins produced by enterohemorrhagic *Escherichia coli* (34, 35, 37, 39–41), hemagglutinin (HA) of IAV (36), or amyloid β (38), a causative factor in Alzheimer’s disease. Here, we customized a tetravalent peptide library to identify high-affinity peptides that bind to a recombinant CaMKII catalytic domain (CaMKII-KD). To increase the basal affinity of the library, we fixed Leu at position −5 (Fig. 1a, upper panel). We chose this position because Leu at −5 from the putative phosphorylation site is highly conserved in substrates and substrates/activators and increases the binding affinity through its interaction with a hydrophobic pocket on the kinase domain comprised of Phe98, Ile101, Val102, and Ile205 (42). We screened the library twice (Fig. 1a, middle panel) and found a strong selection for (i) Leu from position −4 to position −1, (ii) Arg at position –8, (iii) Arg or Ile at positions −7 and −6, and (iv) Arg-His-His, Leu-Leu-His, or Arg-Leu-His motifs from position +1 to position +3. We identified six candidate motifs and synthesized monovalent peptides (M1–M6) and tetravalent peptides (T1–T6) with these motifs as possible inhibitors of CaMKII (Fig. 1a, lower panel).

All of the synthetic peptides except M4, T4, and T5 inhibited the kinase activity of CaMKII-KD efficiently with the indicated IC_50_ values (Fig. 1b), suggesting that the multivalency of the functional motifs did not significantly affect inhibition in this case. Thus, the monovalent peptides were used for the following experiments. In contrast to their effect on CaMKII, the monovalent peptides did not inhibit other representative protein kinases, such as protein kinase C (PKC) or protein kinase A (PKA) (Fig. S1). The kinetics of the binding of these peptides to CaMKII-KD were determined using the BIAcore system. M3 bound most efficiently to CaMKII-KD (K_D_ = 3.4 µM), followed by M1 and M2 (Fig. 1c); however, clear kinetics data were not obtained with the other peptides because of their non-specific binding to the sensor chip.

Next, we determined whether the peptides inhibited CaMKII activation in cells. CaMKII promotes Toll-like receptor (TLR)–triggered proinflammatory cytokine production in macrophages (43). Here, using a RAW264.7 macrophage cell line, we measured the inhibitory effects of the peptides on proinflammatory cytokine production induced by the TLR-4 agonist lipopolysaccharide (LPS) (Fig. S2a). M6 inhibited tumor necrosis factor α (TNFα) production most efficiently, followed by M3 (Fig. 1d, left panel). To increase the cell permeability, we synthesized N-terminal myristoylated-M3 (mM3) and -M6 (mM6) (myr-Met-Ala-[no. 3 or 6 motif]-Ala) and found that mM3 inhibited TNFα production similarly to M6 (Fig. 1d, right panel). Furthermore, M6 and mM3 efficiently inhibited the LPS-induced Thr286 autophosphorylation of CaMKII, which generates Ca^2+^/CaM-independent constitutive activation of CaMKII (44), and the subsequent transcription of interleukin-6 (IL-6), TNFα, and interleukin-1β (IL-1β) (Fig. S2b and c).

### M3 inhibits IAV propagation efficiently in MDCK cells

**Fig 2:**
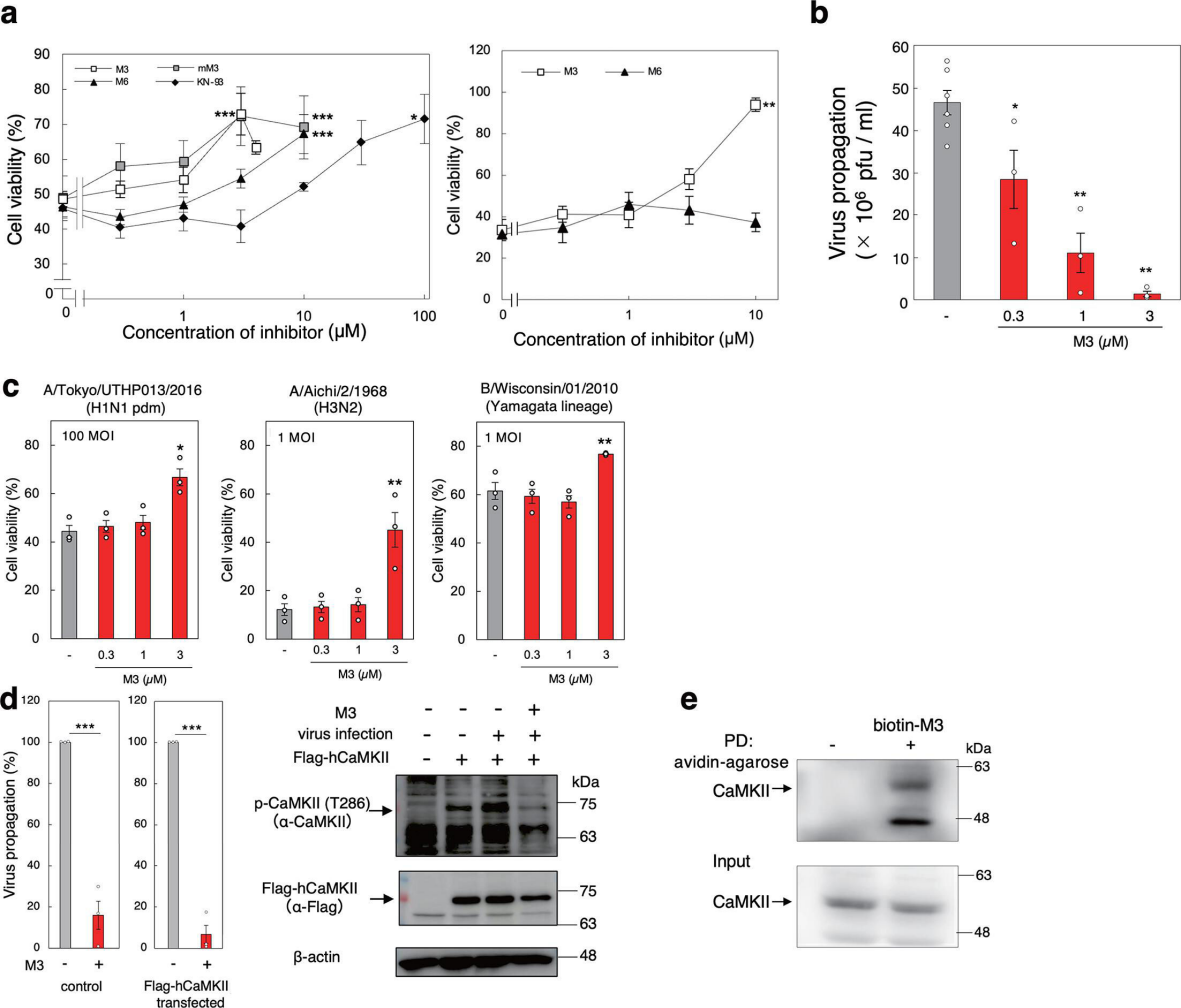
M3 inhibits IAV propagation in MDCK cells. (a) The effects of monovalent peptides on the cytopathicity induced by infection. MDCK cells were treated with peptides for 30 min and then infected with IAV strain PR8 at MOI = 20 (left panel) for 24 h or MOI = 0.001 (right panel) for 40 h. Data are presented as a percentage of the control value without infection (left panel; KN-93: *n* = 3, mM3: *n* = 4, M3: *n* = 10, M6: *n* = 11, virus alone: *n* = 16, right panel; *n* = 3, mean ± SEM). *P < 0.05; **P < 0.01; ***P < 0.001 (compared with the non-treated control cells by ANOVA followed by one-sided Dunnett’s test). (b) The effect of M3 on virus propagation. MDCK cells were incubated with M3 for 30 min and then infected with IAV strain PR8 at MOI = 0.2 for 16 h. The virus titer in the supernatant was determined by a plaque assay. Data are presented as the fold increase over the initial virus titer (60,000 pfu/mL) (*n* = 3–6, mean ± SEM). *P < 0.05; **P < 0.001 (compared with no M3 treatment by ANOVA followed by one-sided Dunnett’s test). (c) The effects of M3 on the cytopathicity induced by infection of other strains. MDCK cells were treated with M3 for 30 min and then infected with IAV strain Tokyo/UTHP013/2016 (H1N1 pdm, MOI = 100), Aichi/2/1968 (H3N2, MOI = 1), or IBV strain Wisconsin/01/2010 (Yamagata lineage, MOI = 1) for 24 h. Data are presented as a percentage of the control value without infection (*n* = 3, mean ± SEM). *P < 0.05; **P < 0.001 (compared with no M3 treatment by ANOVA followed by one-sided Dunnett’s test). (d) The effect of M3 on virus propagation after IAV infection (left panel). MDCK cells transfected with FLAG-tagged hCaMKIIor mock-transfected cells were incubated with or without 3 µM M3 for 30 min prior to infection with IAV strain PR8 at MOI = 0.2 for 16 h. The virus titer in the supernatant was determined by a plaque assay. Data are presented as the fold increase over the initial virus titer (*n* = 3, mean ± SEM). ***P < 0.001 (compared with no M3 treatment by Student’s *t*-test). The effect of M3 on activation of CaMKII induced by IAV infection (right panel). MDCK cells transfected with FLAG-tagged hCaMKII or mock-transfected cells were incubated with 3 µM M3 for 30 min prior to infection with IAV strain PR8 at MOI = 10 for 1 h. The cell lysates were analyzed by western blot using a phosphorylated Ser286-specific antibody or anti-FLAG antibody. Data are representative of the results from three independent experiments. (e) The binding of M3 to intracellular CaMKII. MDCK cells were treated with 3 µM biotinylated M3 for 90 min at 37°C. Cell lysates were incubated with avidin-agarose beads, and coprecipitating proteins were analyzed by western blot. PD, pull down.

The effects of the identified peptides on IAV cytopathicity were examined. Using a single cycle of infection of Madin-Darby canine kidney (MDCK) cells with IAV strain PR8 at a multiplicity of infection (MOI) of 20, we found that M3 and mM3 had the greatest inhibitory effect on IAV cytopathicity at concentrations up to 3 µM (Fig. 2a and Fig. S3, left panels). Cell viability was much greater for M3 and mM3 than for KN-93. For a multi-cycle infection (MOI = 0.001), M3 and M4 showed high levels of virus inhibition (Fig. 2a and Fig. S3, right panels). Therefore, we used M3 to demonstrate the dose-dependent inhibition of IAV propagation in a single cycle of infection of MDCK cells (Fig. 2b). Using a single cycle of infection, we also demonstrated that M3 had broad anti-influenza activity as it inhibited the cytopathicity of two other IAV strains, Tokyo/UTHP013/2016 (H1N1 pdm) and Aichi/2/1968 (H3N2), as well as the type B influenza virus (IBV) Wisconsin/01/2010 (Yamagata lineage), all of which cause annual influenza epidemics (Fig. 2c). We confirmed that M3 had no direct effects on IAV particles because co-incubation of M3 and IAV did not affect the IAV-induced hemagglutination or the virus titer (Fig. S4).

**Fig 3:**
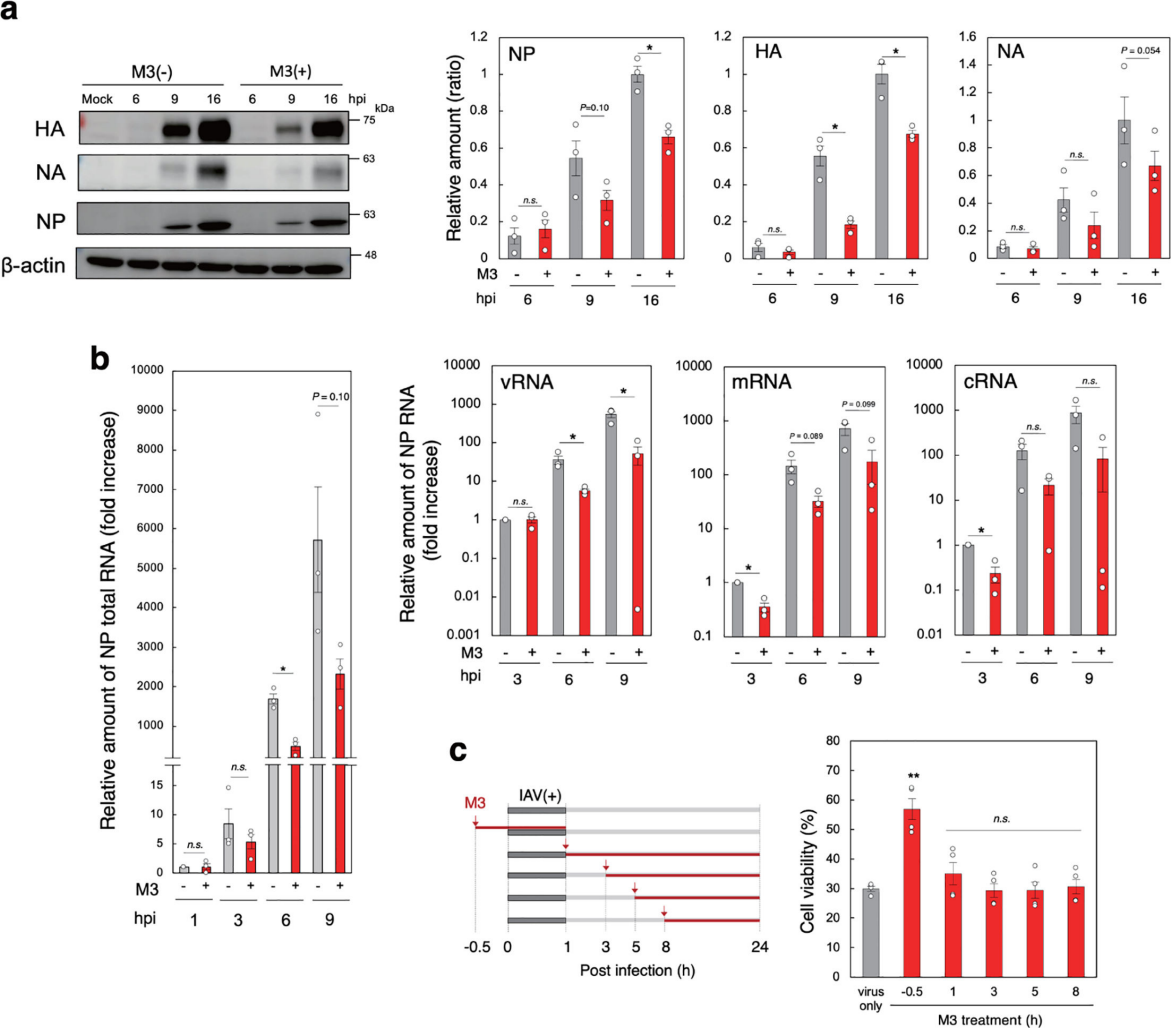
M3 inhibits viral RNA and protein synthesis to reduce virus propagation. (a) The effects of M3 on viral protein synthesis. MDCK cells were incubated with or without 3 µM M3 for 30 min and then infected with IAV strain PR8 at MOI = 1 for the indicated time. Viral proteins in the lysate were analyzed by western blot, and the intensity of each band was quantitated and presented as the amount of each viral protein relative to β-actin (right panels). Data are representative of the results of three independent experiments (left panel) and are presented as a ratio compared to the value at 16 h post-infection without M3 (*n* = 3, mean ± SEM). *P < 0.05 (compared with no M3 treatment by Student’s *t*-test). (b) The effects of M3 on the expression of NP RNA. MDCK cells were incubated with or without 3 µM M3 for 30 min and then infected with IAV strain PR8 at MOI = 1 for the indicated time. The relative amounts of NP total RNA (left panel), vRNA, mRNA, and cRNA (right panels) were determined by reverse transcriptase-quantitative PCR (RT-qPCR) using *GAPDH* as the reference gene. Data are presented as a fold increase over the average RNA level at 1 h (left panel) or 3 h (right panels) post-infection without M3 (*n* = 3, mean ± SEM). *P < 0.05 (compared with no M3 treatment by Student’s *t*-test). (c) The effect of M3 treatment at various time points on the cytopathicity of IAV. MDCK cells were infected with strain PR8 at MOI = 20 for 1 h and then incubated for 23 h. The treatment of 3 µM M3 was performed as shown in the schematic diagram (left panel). Data are presented as a percentage of the control value without infection (right panel, *n* = 4, mean ± SEM). **P < 0.01 (compared with no M3 treatment by ANOVA followed by one-sided Dunnett’s test). *n.s*., not significant. hpi, hour post-infection.

To determine whether M3 inhibited virus propagation by inhibiting CaMKII activation in cells, we measured its effect on the propagation of IAV in MDCK cells expressing FLAG-tagged CaMKII. M3 inhibited IAV propagation in these cells (Fig. 2d, left panel) and inhibited the phosphorylation of the Thr286 of CaMKII, which was enhanced by IAV infection (Fig. 2d, right panel). To determine whether M3 binds directly to CaMKII in cells, we performed coprecipitation assays with biotinylated M3, which revealed that CaMKII coprecipitated with M3 (Fig. 2e). Thus, our results indicate that M3 inhibits IAV propagation through the direct inhibition of CaMKII in host cells.

### M3 inhibits viral RNA synthesis to reduce viral protein production

To determine the mechanism of M3 inhibition of IAV propagation, we measured its effect on the production of viral proteins HA, NA, and nucleoprotein (NP) over time and found the inhibition of all three proteins (Fig. 3a). Next, we measured M3 inhibition of NP total RNA and found a substantial reduction after 6 h of infection by M3 treatment (Fig. 3b, left panel). After entry of IAV into the host cell, the viral ribonucleoprotein (vRNP) complex, which consists of viral RNAs (vRNA), NP, and viral RNA-dependent RNA polymerase, is transported to the nucleus, where viral mRNA (v-mRNA) is produced by the polymerase using parental vRNA as a template (45–47). The v-mRNA is translated into viral proteins, including the viral polymerase, which, in the nucleus, replicates complementary RNA (cRNA) and vRNA, using vRNA and cRNA, respectively, as the templates. Therefore, we measured the effect of M3 on all NP RNAs and found that M3 decreased the amount of both mRNA and cRNA at 3 h post-infection, followed by a marked reduction of vRNA, which is assembled into vRNP complex to produce a new virus particle, indicating that M3 functions early in infection (Fig. 3b, right panels). We determined when M3 was most effective by infecting MDCK cells with IAV for 1 h and then incubating for 23 h. M3 effectively inhibited the cytopathicity in cells treated with M3 during the first hour of incubation with the virus (Fig. 3c). However, cells treated with M3 at later time points beginning 1 h after infection showed little inhibition. On the other hand, M3 did not affect the virus entry process. This was determined not only by a fusion assay using IAV particles labeled with two different lipophilic tracers (Fig. S5a) but also by measuring the total amount of NP vRNA at 1 h post-infection, which reflects the amount of NP vRNA of parental IAV endocytosed into the cells (Fig. S5b). Thus, it appears that M3 targets an early event in infection that is critical for virus propagation via the activation of CaMKII, but not the virus entry process.

### M3 specifically inhibits the early cap-snatching from host mRNA that promotes viral mRNA synthesis

**Fig 4:**
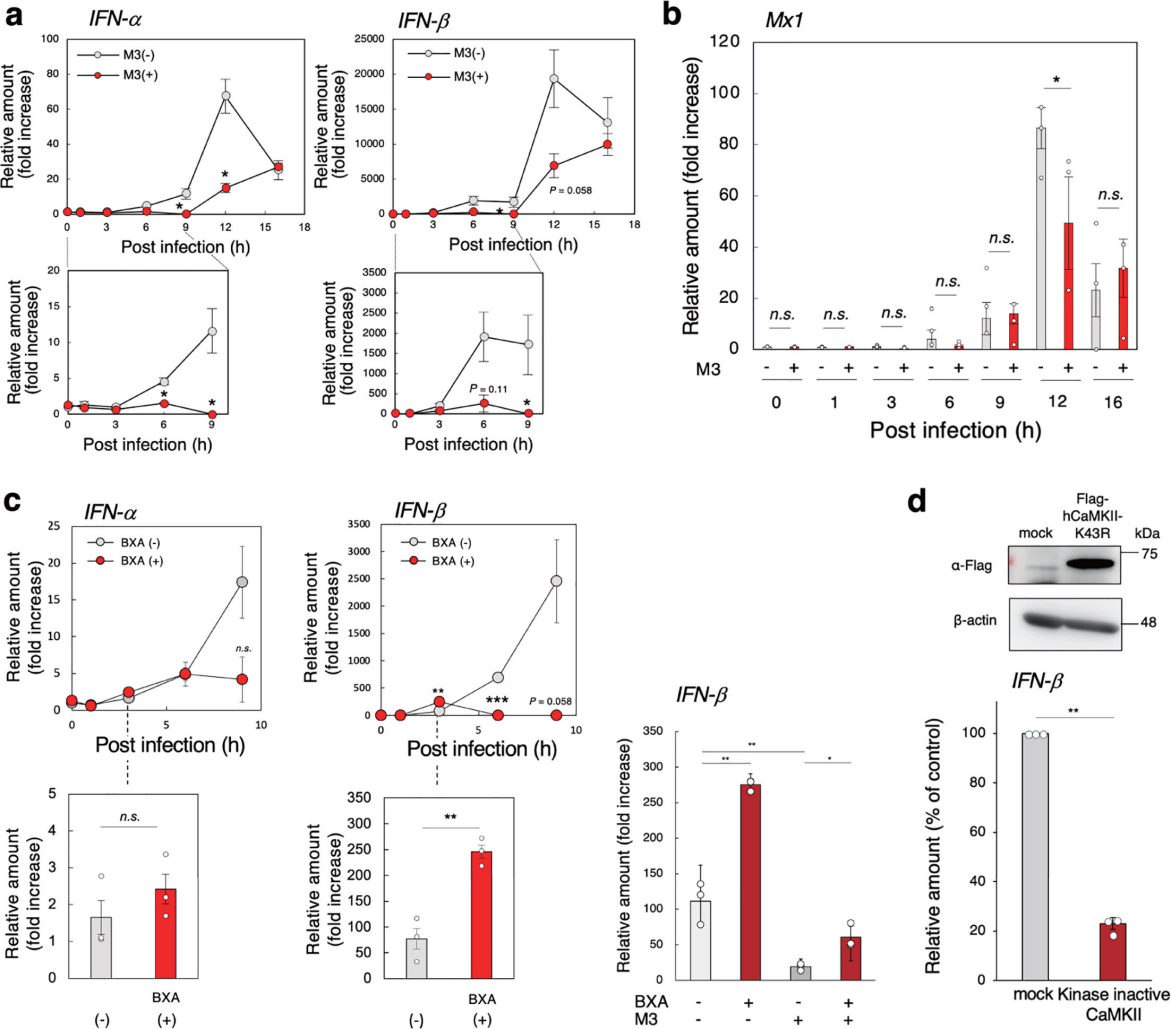
M3 specifically inhibits cap-snatching early in infection. (a, b) The effects of M3 on the expression of mRNAs for IFNα or β (a) or Mx1 (b). MDCK cells were incubated with or without 3 µM M3 for 30 min and then infected with IAV strain PR8 at MOI = 1 for the indicated time. Data up to 9 h after infection are enlarged (a, lower panels). The relative amount of each mRNA was determined by RT-qPCR using *GAPDH* as the reference gene. Data are presented as the fold increase over the average RNA level before infection (*n* = 3, mean ± SEM). *P < 0.05 (compared with no inhibitor by Student’s *t*-test). (c) The effect of BXA on the expression of IFNα and β mRNAs.MDCK cells were incubated with or without 100 nM BXA for 30 min and then infected with IAV strain PR8 at MOI = 1 for the indicated time. Data for 3 h after infection are enlarged (left panels). The relative amount of each mRNA was determined by RT-qPCR using *GAPDH* as the reference gene. Data are presented as the fold increase over the average RNA level before infection (*n* = 3, mean ± SEM). **P < 0.01; ***P < 0.005 (compared with no inhibitor by Student’s *t*-test). The effect of M3 on the enhanced expression of IFNβ mRNA by BXA (right panel). MDCK cells were incubated with 100 nM BXA and/or 3 µM M3 for 30 min and then infected with IAV for 3 h. Data are presented as the fold increase over the average RNA level before infection (*n* = 3, mean ± SEM). *P < 0.05; **P < 0.01 (by Tukey’s test). (d) The effect of overexpression of kinase-inactive Flag-hCaMKII-K43R on the expression of IFNβ mRNA. MDCK cells transfected with Flag-hCaMKII-K43R or mock-transfected cells were infected with IAV strain PR8 at MOI = 1 for 6 h. The cell lysates were analyzed by western blot using anti-FLAG antibody. The relative amount of IFNβ mRNA was determined by RT-qPCR using *GAPDH* as the reference gene, and the fold increase over the average RNA level before infection was determined. Data are presented as a percentage of the control value (*n* = 3, mean ± SEM). **P < 0.01 (compared with mock by Student’s *t*-test).

Type I IFNα and β act as major antiviral proteins through the expression of a series of ISGs, such as Mx1 (30). Infection with IAV markedly induces the expression of IFNα and β mRNAs, which peaks at 12 h after infection, reflecting the activation of the RIG-I pathway to transduce antiviral signaling (28, 29). When we measured the effect of M3 on IFNα and β mRNAs, we unexpectedly found decreased expression of both mRNAs (Fig. 4a, upper panels). In particular, M3 completely abolished the expression up to 9 h after infection (Fig. 4a, lower panels), whereas induction levels at 16 h after infection were similar with or without M3. Also, we saw substantial expression of Mx1 mRNA 16 h after infection, even in the presence of M3 (Fig. 4b). These results indicate that there is an alternative pathway that induces the expression of IFNα and β mRNAs at an early stage of infection, distinct from the canonical RIG-I pathway, which functions later in infection. This pathway, which is highly sensitive to M3 treatment, may play an essential role in efficient virus propagation.

To elucidate this alternative pathway in virus propagation, we determined whether the IFNα and β mRNAs expressed early in infection can be a source of the m7G caps that are provided for viral mRNA transcription through the cap-snatching mechanism (31, 32). Baloxavir acid (BXA), a therapeutic influenza drug, suppresses virus propagation by inhibiting the cap-dependent endonuclease activity of viral PA, which functions as a component of the viral RNA-dependent RNA polymerase complex to provide the m7G caps (48). Under conditions in which BXA efficiently inhibited the infection-induced expression of NP mRNA and cytopathicity (Fig. S6), BXA also greatly reduced the massive levels of expression of IFNα and β mRNAs later in infection (Fig. 4c, left upper panels). However, early in infection, BXA rather increased the expression of both mRNAs (Fig. 4c, left lower panels). Therefore, the IFNα and β mRNAs expressed early in infection can be an essential source of m7G caps to stimulate viral mRNA synthesis, thereby enhancing the synthesis of cRNA and vRNA for virus propagation. Later in infection, these viral RNAs are sensed by the RIG-I pathway to induce the massive expression of IFN mRNAs. Consistently, the enhanced expression of IFNβ mRNA by BXA after 3 h of infection was markedly inhibited by M3 treatment (Fig. 4c, right panel). Thus, M3 markedly inhibited the initial expression of IFN mRNAs as a source of the caps, thereby inhibiting virus propagation. However, IFN mRNA levels were much lower early than later in infection. Furthermore, the initial expression of IFNβ mRNA was suppressed by the overexpression of the kinase inactivated CaMKII mutant (Fig. 4d), clearly indicating that CaMKII is involved in this event.

In addition to type I IFNs, mRNA expression of proteins related to antiviral responses, such as CXCL10, IFIT1, and CCL5, are upregulated after infection (32). Early in infection, M3 decreased the expression of CXCL10 and CCL5 mRNAs (Fig. S7a), whereas BXA also increased their expression (Fig. S7b), indicating that these mRNAs, which are targets for M3, can be a source of m7G caps. Thus, CaMKII can provide the caps essential for virus propagation, especially early in infection, by inducing the expression of a wide range of mRNAs, including mRNAs of type I IFNs and proinflammatory cytokines.

### A non-canonical RIG-I pathway regulated by CaMKII plays an essential role in virus propagation

**Fig 5:**
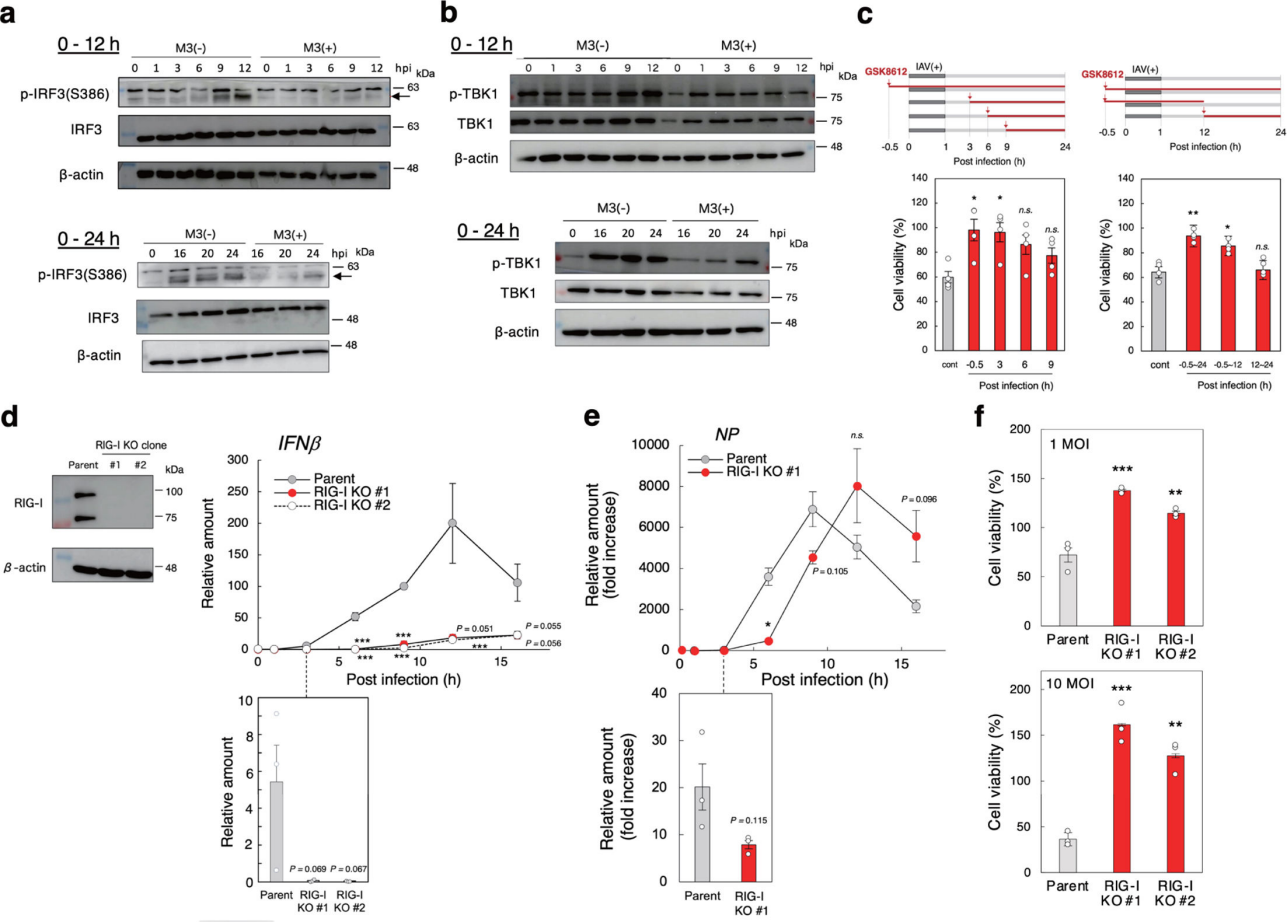
M3 inhibits a non-canonical RIG-I pathway that acts early in infection to promote virus propagation. (a, b) The effects of M3 on the activation of IRF3 (a) or TBK1 (b) induced by the infection. A549 cells were incubated with or without 3 µM M3 for 30 min, and then infected with IAV strain PR8 at MOI = 1 for the indicated time. The cell lysates were analyzed by western blot, and the data are representative of the results from three independent experiments. (c) The effects of the TBK1 inhibitor GSK8612 on the cytopathicity induced by infection. MDCK cells were infected with IAV strain PR8 at MOI = 10 for 1 h and then incubated for 23 h with 10 µM GSK8612 treatment during the time periods shown in the upper panels. Data are presented as the percentage of the control value without infection (*n* = 4, mean ± SEM). *P < 0.05; **P < 0.01 (compared with no GSK8612 treatment by ANOVA followed by one-sided Dunnett’s test). (d) The effects of the *RIG-I* KO on the IFNβ mRNA expression. The expression of RIG-I in MDCK-derived *RIG-I* KO clones was determined by western blot. The upper and lower bands represent full-length RIG-I and its spliced variant, respectively (left panel). Cells of parental MDCK or both MDCK-derived *RIG-I* KO clones were infected with IAV strain PR8 at MOI = 1 for the indicated time (right panel). Data for 3 h after infection are shown in the lower panel. The relative amount of IFNβ mRNA was measured by RT-qPCR using *GAPDH* as the reference gene, with the amount at 9 h post-infection in parental cells equal to 100 (*n* = 3, mean ± SEM). ***P < 0.005 (compared with the control cells by ANOVA followed by one-sided Dunnett’s test). (e) The effect of the *RIG-I* KO on NP mRNA expression. Cells of MDCK or MDCK-derived *RIG-I* KO clone #1 were infected with IAV strain PR8 at MOI = 1 for the indicated time. The relative amount of NP mRNA was measured by RT-qPCR using *GAPDH* as the reference gene. Data are presented as the fold increase over the average RNA level before infection (*n* = 3, mean ± SEM). *P < 0.05 (compared with no inhibitor by Student’s *t*-test). (f) The effects of the *RIG-I* KO on the cytopathicity induced by infection. Cells of parental MDCK or the two MDCK-derived *RIG-I* KO clones were infected with IAV strain PR8 at MOI = 1 for 24 h. Data are presented as a percentage of the control value without infection (*n* = 3, mean ± SEM). *P < 0.05; **P < 0.01; ***P < 0.001 (compared with parental MDCK by ANOVA followed by one-sided Dunnett’s test).

The expression of mRNAs encoding IFNα, IFNβ, and various proinflammatory cytokines is induced by the activation of IRF 3/7 (30) and partially by NF-κB. To understand the CaMKII-dependent regulation of the expression of IFNα and β mRNA early in infection, we focused on IRF3 activation because the expression of IRF7 mRNA is regulated by IFN (49, 50). Consistently, we found that IRF7 mRNA expression, but not IRF3 mRNA, was induced after 6 h of infection and was reduced by M3, further confirming that IRF7 activation is regulated by IFN (Fig. S8). The effect of M3 on the activation of IRF3 was determined in A549 human lung adenocarcinoma epithelial cells. In A549 cells, the IAV-induced cytopathicity was efficiently inhibited by M3 (Fig. S9a) and knockdown of CaMKII using siRNA (Fig. S9b). In addition, IFNβ expression early in infection was inhibited by M3 (Fig. S9c) and knockdown of CaMKII (Fig. S9d). M3 efficiently inhibited the infection-induced activation of IRF3, which is caused by the phosphorylation of Ser386 in IRF3 by TBK1 in both early and later stages of infection (Fig. 5a). Also, the activated, phosphorylated form of TBK1 was inhibited by M3 with a similar temporal pattern (Fig. 5b). The TBK1 inhibitor GSK8612 (51) inhibited the IAV-induced cytopathicity of MDCK cells best when GSK8612 was added up to 3 h after infection (Fig. 5c, left panel), and treatment given only early in infection (−0.5 h to 12 h) was also effective (Fig. 5c, right panel). This confirmed that the induction of IFN mRNAs early in infection through TBK1 and IRF3 activation is essential for virus propagation.

The activation of TBK1 and IRF3 is conventionally induced by the canonical RIG-I pathway later in infection (28, 29). To determine whether the IFN mRNA expression induced by TBK1 and IRF3 activation early in infection is regulated by RIG-I, we measured the effects of knocking out *RIG-I*. We confirmed the loss of RIG-I in the MDCK-derived knockout (KO) clones (Fig. 5d, left panel). Notably, IAV-induced IFNβ mRNA expression was markedly reduced both early (3–6 h after infection) and later in infection (Fig. 5d, right panel), indicating that RIG-I is also involved in IFNβ mRNA expression at an early stage as well as a later stage of infection. Consistent with this, the expression of viral NP mRNA was significantly reduced early in infection in the *RIG-I* KO clone (Fig. 5e). However, 12 h after infection, the NP mRNA expression surpassed the level of the parental cells, confirming previous observations that the canonical RIG-I pathway inhibits viral production (28, 29). Importantly, IAV-induced cytopathicity was strongly inhibited in these *RIG-I* KO clones compared with parental cells (Fig. 5f), indicating that the non-canonical RIG-I dependent pathway, which is regulated by CaMKII early in infection, supports virus propagation—in contrast to the antiviral canonical RIG-I pathway.

To further elucidate the involvement of RIG-I in the antiviral function of M3, the effect of M3 on the IFNβ mRNA expression induced by 5ʹ triphosphate hairpin RNA (3p-hpRNA), which can directly bind to and activate RIG-I, was examined using A549 cells. The IFNβ mRNA expression induced by 3p-hpRNA was efficiently inhibited by M3 (Fig. S10a) and substantially inhibited by CaMKII knockdown (Fig. S10b). These observations further confirm that M3 directly affects the non-canonical RIG-I pathway through CaMKII inhibition.

### M3 protects mice from the lethal IAV infection

**Fig 6:**
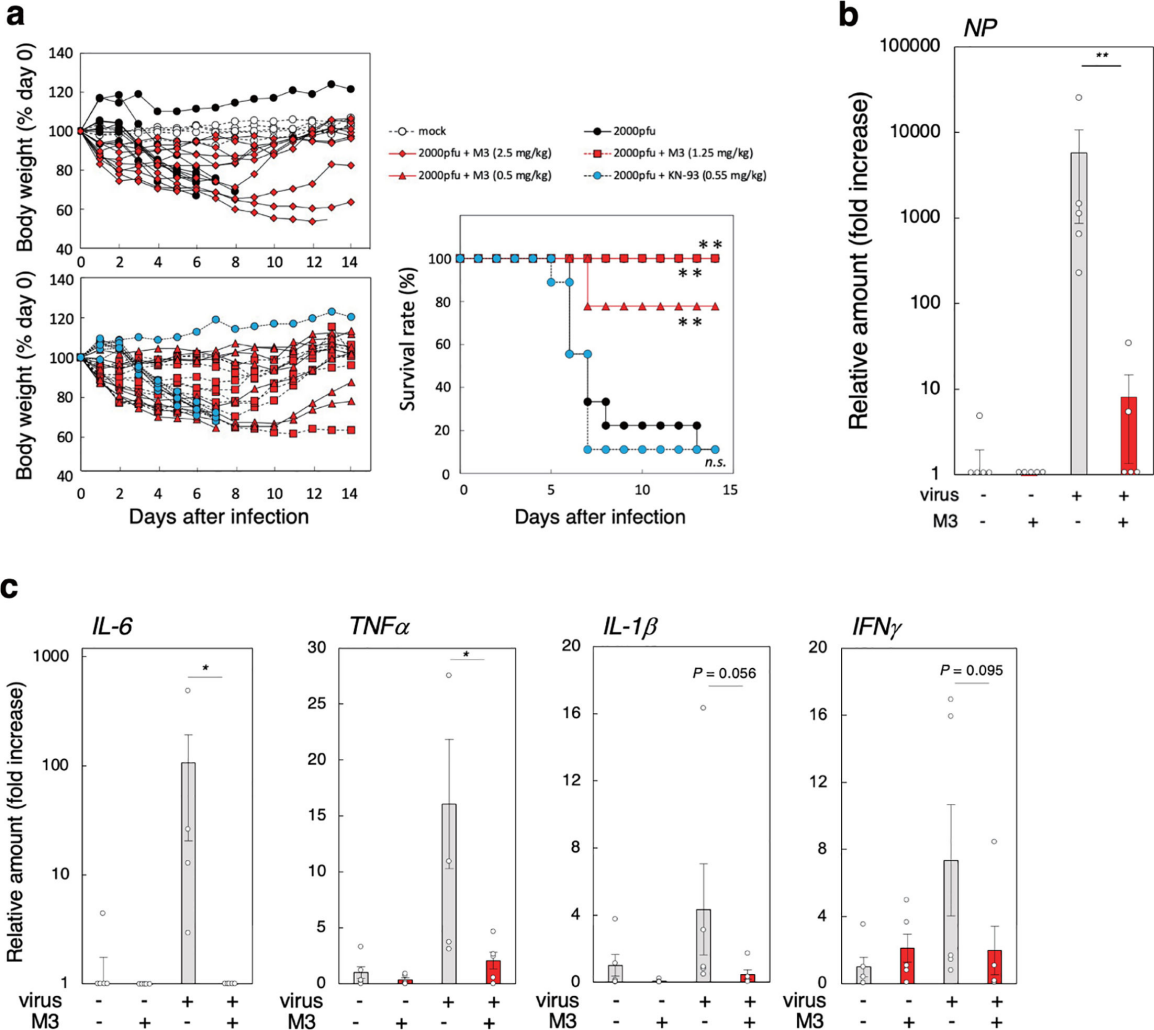
M3 rescues mice from the lethality of IAV infection. (a) The effects of M3 on the lethality of IAV infection. Female BALB/c mice were infected intranasally with 2,000 plaque-forming units (pfu) of IAV strain PR8 with or without 0.5, 1.25, or 2.5 mg/kg M3, or 0.55 mg/kg KN-93. The body weight of each mouse is presented as a percentage of the value on day 0 (left panels), and the survival rate of each group is shown in the right panel. Each group contained 5–9 mice. **P < 0.01 (by log-rank test). (b, c) The effects of M3 on the expression of viral and host mRNAs in the lung after infection. Female BALB/c mice were infected intranasally with 2,000 pfu of IAV strain PR8 with or without 1.25 mg/kg M3. The relative amounts of viral NP mRNA (b) and various proinflammatory cytokine mRNAs (c) in lungs harvested 3 days after infection were determined by RT-qPCR using *GAPDH* as the reference gene. Data are presented as the fold increase over the average RNA level of the control mice (*n* = 5, mean ± SEM). *P < 0.05; **P < 0.01 (by Mann–Whitney U test).

Female 6- to 8-week-old BALB/c mice were intranasally infected with the mouse-adapted IAV strains PR8 (2,000 pfu, equivalent to 10 LD_50_ values) in the presence or absence of the indicated amount of M3 or KN-93. Intranasal infection of mice with IAV caused acute weight loss from day 3 after infection and 90% mortality within 14 days. Intranasal co-injection of 0.5, 1.25, or 2.5 mg/kg M3 rescued 78%, 100%, or 100% of mice from mortality, respectively, and partially attenuated the weight loss (Fig. 6a). Under the same conditions, 0.55 mg/kg KN-93 (equivalent to 2.5 mg/kg M3 on a molar basis) provided no protection. The increase in viral NP mRNA expression in the lung 3 days after infection was markedly reduced by 2.5 mg/kg M3 treatment (Fig. 6b). Together, these results indicate that M3 has significant anti-IAV activity *in vivo*.

The production of large amounts of various proinflammatory cytokines in the lung after IAV infection that result in cytokine storms is a major cause of lethality (52). We found that 2.5 mg/kg M3 treatment greatly reduced proinflammatory cytokine (IL6, IFNγ, TNFα, and IL-1β) mRNA expression in the lung 3 days after infection (Fig. 6c), which explains the high efficacy of M3 in protecting mice from lethal IAV infection.

## DISCUSSION

**Fig 7:**
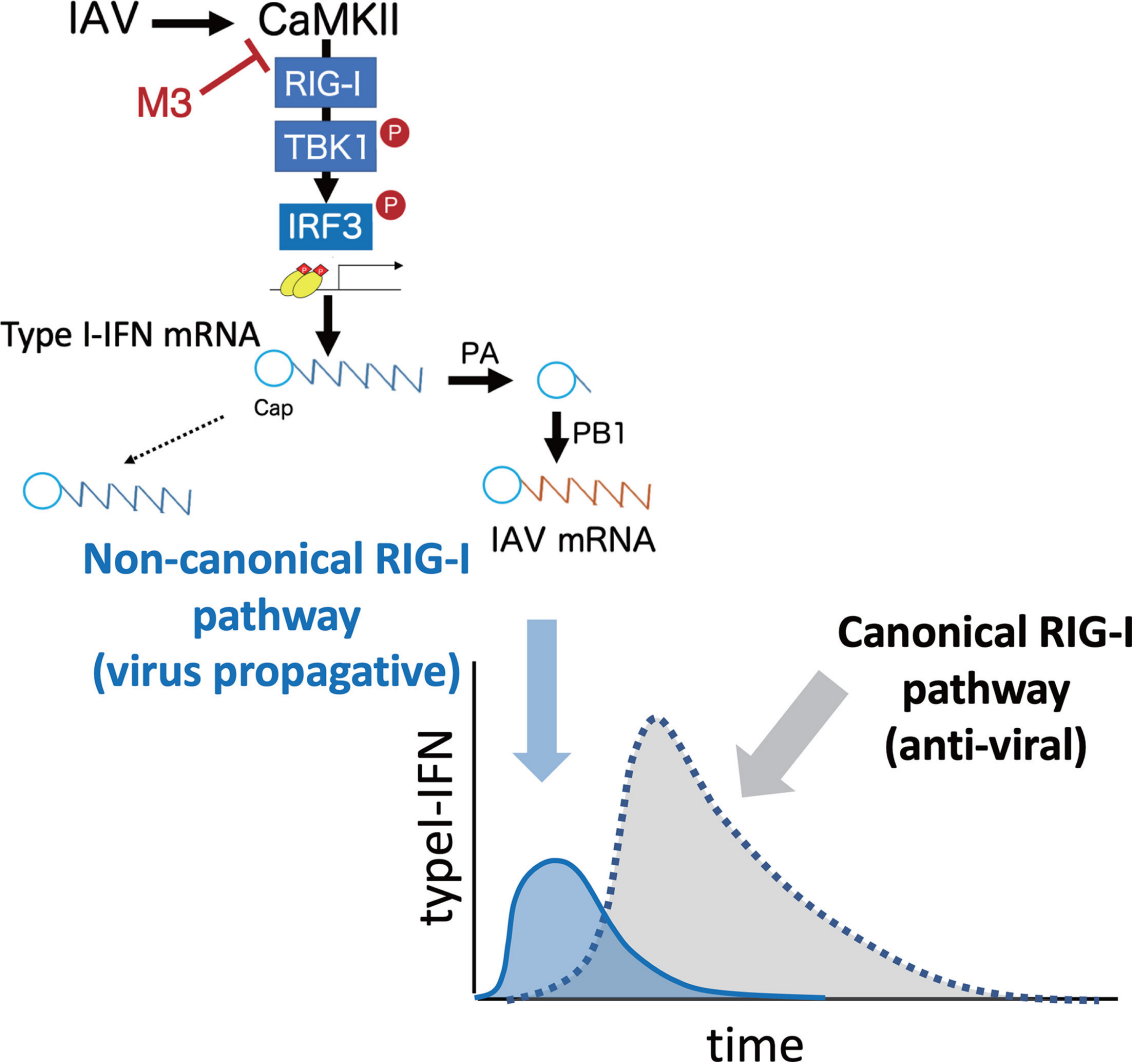
M3 inhibits CaMKII-dependent activation of the non-canonical RIG-I pathway that enhances IAV propagation. At an early stage of IAV infection, CaMKII activates a non-canonical RIG-I pathway to activate TBK1 and IRF3 and then induces small but sufficient amounts of IFN α/β mRNAs whose capped 5′-ends are used preferentially to promote viral mRNA transcription through the cap-snatching mechanism. This pathway acts early in infection in an opposite manner compared with the canonical RIG-I pathway, which functions later in infection to induce high levels of mRNA for IFN α/β and then for antiviral proteins. M3 efficiently suppresses virus propagation by inhibiting the CaMKII-depenent activation of the non-canonical RIG-I pathway at the early stage of infection.

In this study, we identified M3 as a novel CaMKII inhibitory peptide by targeting the catalytic domain using affinity-based screening of a random peptide library customized for CaMKII. Our studies with M3 revealed that early in IAV infection, CaMKII activates a novel non-canonical RIG-I pathway that, in turn, activates TBK1 and IRF3, which induce transcription of the genes for IFN α/β and proinflammatory cytokines. The capped 5′-ends of these transcripts are used preferentially to enhance virus propagation via the cap-snatching mechanism. Thus, this pathway acts in an opposite manner compared with the canonical RIG-I pathway, which functions later in infection to induce high levels of mRNA for IFN α/β and then for several antiviral proteins (Fig. 7). Inhibition of CaMKII by M3 during only the first hour after infection strongly inhibited the cytopathicity of infection (Fig. 3c), indicating that this pathway provides a promising target for the treatment of influenza.

Here, we identified six high-affinity binding motifs to the catalytic domain of CaMKII that shared the sequence Arg-Arg/Ile-Arg/Ile-Leu-Leu-Leu-Leu-Leu-Ala-Arg/Leu-His/Leu-His. Notably, the sequences are substantially different from the predicted optimal substrate motif for CaMKII, as determined by a peptide library screening (53), a representative CaMKII substrate peptide GluN2B (NMDA receptor peptide substrate 1289–1310) (42), or previously identified CaMKII inhibitory peptides (24), such as AIP (54) and CaMKIINtide (24, 55)—all sequences that are very similar to each other (Fig. S11). Focusing on M3, we characterized the interaction of M3 with the CaMKII catalytic region using molecular dynamics simulations (Fig. S12). In five independent trials, we observed strong interactions between the catalytic cleft of CaMKII-KD and the entire functional region of M3. The binding free energy of the interaction between the catalytic cleft and M3 (−166.94 kcal/mol) was even lower than with CaMKIINtide (−146.78 kcal/mol). In particular, the (−3)Leu of M3 interacts stably with Phe98 of CaMKII-KD through hydrophobic interaction (Fig. S12b), whereas the (−3)Arg of CaMKIINitde interacts electrostatically with Glu96 of CaMKII-KD (42). The (−5)Ile, which corresponds to the (−4)Leu of M3, and (−2)Ser of CaMKIINitde form hydrophobic interactions with P210 and Trp213 and hydrogen bonds with Glu139 and Lys137, respectively. In contrast, the (−4)Leu and (−2)Leu of M3 fully occupy a hydrophobic pocket comprised of Tyr179, P211, Phe213, Trp214, and Leu221, further indicating a unique and effective inhibitory interaction of M3 with CaMKII-KD.

Here, we found a non-canonical RIG-I pathway that is activated by CaMKII early in infection to enhance virus propagation through cap-snatching. Cells with a *RIG-I* KO showed decreased expression of IFN mRNAs and subsequent NP mRNA early in infection, which then protected cells from cytopathicity 24 h after infection. On the other hand, later in infection, a *RIG-I* KO abolishes the antiviral activity of the canonical RIG-I pathway, which induces high levels of IFN mRNA expression and then causes the expression of various antiviral proteins, resulting in the induction of delayed but sufficient NP mRNA (Fig. 5e) to support virus propagation (Fig. S13). Previous studies using *RIG-I* KO mice also demonstrated that RIG-I is critical for protection against IAV infection, including the clearance of IAV from the lung through the enhanced expressions of IFNβ (56, 57). In contrast to the *RIG-I* KO cells, M3 treatment of cells specifically inhibited the CaMKII-dependent RIG-I pathway early in infection without affecting the canonical RIG-I pathway. This resulted in highly efficient inhibition of not only the cytopathicity induced by the infection but also virus propagation. The importance of this novel RIG-I pathway in supporting virus propagation was confirmed when the loss of this pathway by *RIG-I* KO almost completely inhibited NP mRNA expression up to 6 h after infection (Fig. 5e), which has not been reported previously.

Previously, it has been shown that the TLR3/4 pathway causes CaMKII activation to induce the mRNA expression of inflammatory cytokines, such as IL-6, TNFα, and IFNα/β in macrophages (43). However, in this study, the enhanced IFNβ mRNA expression observed early in infection, which was markedly suppressed by M3 and overexpression of kinase-inactive CaMKII mutant (Fig. 4a and d), was also significantly inhibited by RIG-I knockout (Fig. 5d). Furthermore, IFNβ mRNA expression induced by 3p-hpRNA, which can specifically activate RIG-I, was efficiently inhibited by M3 and CaMKII knockdown (Fig. S10). These observations clearly indicate that the RIG-I pathway (non-canonical RIG-I pathway), but not the TLR3/4 pathway, is involved in the enhanced IFNβ mRNA expression early in infection.

At present, it is not clear how CaMKII activates RIG-I early in infection. Activation of RIG-I by a series of post-translational modifications (58) begins with the recognition of viral dsRNA through the C-terminal RNA interaction domain (CTD) that induces a conformational change in RIG-I. Subsequently, dephosphorylation of Ser8 and Thr170 in the two N-terminal caspase activating and recruiting domains (CARD), and Ser854, Ser855, and Thr770 in the CTD by protein phosphatases (PPase), such as PP1-α/γ (59), stimulate the ubiquitination of each domain by their respective E3 ubiquitin ligases, enhancing their interaction with MAVS that transduce the downstream signaling (58). The most important difference between the novel CaMKII-dependent RIG-I pathway and the canonical RIG-I pathway is the amount of viral RNAs sensed by RIG-I. The amount of NP RNA at 3 h after infection, when IFNβ mRNA provided a sufficient source of mRNA for cap-snatching (Fig. 4c), was less than 1% of the amount at 6 or 9 h after infection (Fig. 3b). LGP2, another RLR family member, which lacks any CARDs and was originally identified as a negative regulator of RLR signaling, facilitates vRNA recognition by RIG-I through its ATPase domain (60). Thus, it is possible that CaMKII is involved in the LGP2-dependent process by enhancing the sensing of low amounts of viral RNAs by RIG-I. Another possibility is that activated CaMKII enhances F-actin remodeling, as reported previously (61), and then activates RIG-I by inducing its dephosphorylation. This idea is based on the recent observation that several RNA viruses, including IAV, result in the relocalization of PPP1R12C, a regulatory subunit of PP1-α/γ, from filamentous actin to the cytosol through actin remodeling to allow activation of PP1-α/γ followed by dephosphorylation-mediated priming of RIG-I (62). However, a better understanding of the precise mechanisms by which CaMKII activates the RIG-I pathway remains to be elucidated.

Coadministration of M3, but not KN-93, with IAV infection rescued mice from the lethality of IAV PR8 infection, indicating that M3 is highly effective against IAV *in vivo*. In addition, M3 also efficiently inhibited the cytopathicity induced by other IAV strains, Tokyo/UTHP013/2016 (H1N1 pdm) and Aichi/2/1968 (H3N2), or IBV Wisconsin/01/2010 (Yamagata lineage). RIG-I signaling is also essential for IBV (B/Shangdong/7/97)-induced IRF3 activation and subsequent IFN mRNA expressions in mouse embryonic fibroblasts, occurring earlier and faster than for IAV infection (63). Thus, M3 may act similarly against IBV and IAV. Our finding that M3 is a novel CaMKII inhibitory peptide and our discovery of the non-canonical CaMKII-dependent RIG-I pathway could provide a new strategy to treat infections with various types of influenza viruses.

## MATERIALS AND METHODS

### Antibodies

Antibodies were obtained from the vendors and used at the indicated dilution as shown in Table S1 in the supplemental material.

### Cell culture experiments

All cell lines were cultured in accordance with the supplier’s recommendation (see Table S2).

### Preparation of recombinant CaMKII-KD

Recombinant histidine-tagged CaMKII-KD was expressed in Sf21 cells using baculovirus as follows. A DNA fragment encoding CaMKII-KD was obtained from cDNA of a full length of rat CaMKII by PCR using specific primers (Table S3) and then cloned into a pBacPAK8 transfer vector (Clontech). Recombinant baculovirus was generated using the BacPAK Baculovirus Expression System (Clontech). Sf21 cells were infected with the recombinant baculovirus, and CaMKII-KD was prepared as described previously (64).

### Peptides and library screening

Tetravalent peptide libraries, peptide monomers, and tetravalent peptides were synthesized using N-α-Fmoc-protected amino acids and standard BOP/HOB coupling chemistry as described previously (34, 65). Recombinant CaMKII kinase domain (0.2 mg) bound to Ni^2+^-sepharose beads were incubated with 120 µg of a given library peptide, and the bound peptides were eluted with 30% acetic acid and sequenced on an Applied Biosystems model 477A protein sequencer. The molar ratio of each amino acid recovered from each degenerate position was calculated. The sum of each ratio was normalized to 19 (the number of total amino acids) to evaluate the relative amino acid preference at each degenerate position. Each amino acid would have a value of 1 in the absence of selectivity.

### Kinase assay

Autocamtide-2 peptide (Lys-Lys-Ala-Leu-Arg-Arg-Gln-Glu-Thr-Val-Asp-Ala-Leu), which was synthesized as described above, was phosphorylated by CaMKII-KD in the presence or absence of the indicated amount of inhibitory peptides in the kinase buffer (10 mM HEPES-NaOH (pH 7.5), 2 mM MgCl_2_, 0.2 mM EGTA, 4 mM β-glycero phosphate, 0.004% NP40, 0.2 mM DTT, 20 µM ATP, 2 µCi [γ-^32^P]ATP) for 5 min at 37°C. The amount of radioactivity incorporated into the substrate peptide was determined using the phosphocellulose assay as described previously (66).

### Kinetics analysis of the binding between peptides and CaMKII-KD

The binding of inhibitory peptides to immobilized recombinant CaMKII-KD was quantitated using a BIAcore T100 system instrument (GE Healthcare Sciences, USA) as described previously (36). Purified His-tagged CaMKII-KD (20 µg/mL) was injected into the system and fixed on the Ni^2+^-chelate sensor chip. The resonance unit (RU) is an arbitrary unit (AU) used by the BIAcore system. Binding kinetics were analyzed using BIAevaluation software, v1.1.1 (GE Healthcare Sciences).

### ELISA to measure cytokine production

RAW264.7 cells were incubated with each peptide for 30 min and then treated with LPS (10 ng/mL) for 24 h. The culture medium was applied onto each well of a 96-well enzyme-linked immunosorbent assay (ELISA) plate and incubated for 24 h. After blocking, TNFα production was measured using BD OptEIA™ Mouse TNF ELISA Set II (BD Biosciences, Heidelberg, Germany, Cat#558534).

### Virus preparation

IAV strain A/Puerto Rico/8/1934 (PR8), mouse-adapted IAV strain A/Tokyo/UTHP013/2016 (H1N1 pdm), mouse-adapted A/Aichi/2/1968 (H3N2), and mouse-adapted type B influenza virus (IBV) B/Wisconsin/01/2010 (Yamagata lineage) were prepared as described previously (67). These viruses (H1N1 pdm, H3N2, and IBV) were kindly provided by Drs. Yoshihiro Kawaoka and Mutsumi Ito (Division of Virology, Institute of Medical Science, University of Tokyo, Japan).

### Cytopathicity assay

Confluent MDCK cell monolayers cultured on a 96-well plate were incubated with various concentrations of compounds at 37°C for 30 min and then infected with IAV strain PR8 at 20 or 0.001 MOI. At the indicated time periods, the relative numbers of living cells were determined using cell count reagent SF (Nacalai tesque) according to the manufacturer’s instructions. KN-93, Baloxavir acid, and GSK8612 were purchased from Wako, Shionogi Inc, Sigma-Aldrich, respectively.

### Measurement of virus propagation

Confluent MDCK cell monolayers cultured on a 24-well plate were incubated with various concentrations of M3 at 37°C for 30 min. The cells were infected with IAV at 0.2 MOI in Trypsin(-)-MEM for 16 h. The culture medium was used to determine of the virus titer using a regular plaque-forming assay using the MDCK cell monolayers (67).

### Transfection

A construct for the Flag-tagged human CaMKIIβ was obtained from VectorBuilder Inc. (IL, USA). A kinase-inactive mutant construct with an amino-acid substitution of Lys to Arg (Flag-hCaMKII-K43R) was prepared by PCR. Transfection of the plasmid into MDCK cells was performed using Cell Line Nucleofector™ Kit L (Lonza, Basel, Switzerland) according to the manufacturer’s instructions.

### Coprecipitation assay using biotinylated M3

For coprecipitation from cell lysates, MDCK cells cultured on a 10 cm dish were incubated for 30 min at 37°C in the presence or absence of biotinylated M3 (3 *µ*M). The cell lysates were treated with avidin-agarose beads (Sigma-Aldrich) for 1 h at 4°C. After extensive washing, the beads were analyzed by western blot.

### Western blot

Western blot analysis was performed as described previously (36). See Table S1 for antibodies.

### Quantitative polymerase chain reaction (qPCR)

After harvesting MDCK cells, total RNA was extracted using the FastGene RNA Premium Kit (NIPPON Genetics Co., Ltd, Tokyo, Japan), and transcribed into cDNA using the ReverTra Ace® qPCR RT Master Mix (TOYOBO, Osaka, Japan) according to the manufacturer’s protocols. For the reverse transcription of NP vRNA, NP cRNA, or NP mRNA, a tag sequence specific for each RNA (Table S3) was used as described previously (68). Polymerase chain reaction (PCR) was performed for 40 cycles using the obtained cDNA as a template and specific primers (Table S3). The mRNA levels of each gene were quantified by RT-qPCR using THUNDERBIRD® Next SYBR® qPCR Mix (TOYOBO) and the same primers. Data were analyzed by relative quantification based on the ddCt methods using *GAPDH* as the reference gene, and expressed as the fold increase over the average mRNA levels of non-treated control cells.

### Establishment of MDCK cell clones

MDCK-derived knockout clones of *RIG-I* were prepared using CRISPR-Cas9 genome editing (69). For the construction of sgRNA-Cas9 co-expression vector, DNA fragments (Table S3) were inserted into pSpCas9(BB)−2A-GFP (px458) vector according to the original protocols. px458 vector (Addgene, MA, USA, plasmid #48138) was a gift from Feng Zhang (69). MDCK cells were transfected with sgRNA-Cas9 co-expression vector. After 72 h, EGFP-positive cells were sorted into 96-well plates using an FACSAria II cell sorter (BD Biosciences). Each clone was screened based on protein expression analyzed by western blot using specific antibodies against RIG-I.

### Infection of mice with IAV

Female 6- to 8-week-old, specific pathogen-free BALB/c mice (Shimizu Laboratory Supplies, Japan) were intranasally infected with IAV strains PR8 (2,000 pfu, equivalent to 10 LD_50_ values) in the presence or absence of the indicated amount of M3 or KN-93. The survival rate was analyzed by Kaplan-Meier survival analysis. Under the same condition, the lung was harvested 3 days after infection and homogenized. After centrifugation, the obtained supernatant was used for the measurement of the expression levels of viral and host mRNAs by qPCR.

### Statistical analysis and general methods

Significant differences between the two groups were analyzed using unpaired two-sided Student’s *t*-test or Welch’s *t*-test. Multiple comparisons of differences among every group were analyzed using a one-way analysis of variance (ANOVA) followed by Tukey’s test. Significant differences between each group and the control group were analyzed using one-way ANOVA followed by Dunnett’s test. The non-parametric Mann–Whitney U test was also used to analyze significant differences in data distribution between the two groups. Significant differences in survival rates were analyzed using the log-rank test. All statistical analysis was performed using IBM SPSS Statistics software (ver. 28.0.0.0).

## Data Availability

All data that support the findings of this study are available upon request. The source data for all figures and supplemental figures are provided as source data files in the supplemental material.

## SUPPLEMENTAL MATERIAL

Data Set S1 mbio.00087-24-s0001.xlsx Source data file for Fig. 1 to 4.

Data Set S2 mbio.00087-24-s0002.xlsx Source data file for Fig. 5 and 6.

Data Set S3 mbio.00087-24-s0003.xlsx Source data file for Fig. S1 to S10 and S13.

Supplemental Material mbio.00087-24-s0004.docx Figures S1 to S13; Tables S1 to S3.

ASM does not own the copyrights to Supplemental Material that may be linked to, or accessed through, an article. The authors have granted ASM a non-exclusive, world-wide license to publish the Supplemental Material files. Please contact the corresponding author directly for reuse.
