# Supplementary material for: CaMKII-dependent non-canonical RIG-I pathway promotes influenza virus propagation in the acute-phase of infection
Source: mBio. 2024 Nov 27;16(1):e00087-24. doi: 10.1128/mbio.00087-24 (PMC11708044; doi:10.1128/mbio.00087-24)
Supplement: Supplemental Material — Figures S1 to S13; Tables S1 to S3. [file mbio.00087-24-s0004.docx]

**Supporting Information for**

**CaMKII-dependent non-canonical RIG-I pathway promotes influenza virus propagation in the acute-phase of infection.**

Shinichiro Hama^1^, Miho Watanabe-Takahashi^1^, Hiroki Nishimura^1^, Jumpei Omi^2^, Masakazu Tamada^1^, Takashi Saitoh^3^, Katsumi Maenaka^4,5,6,7^, Yuta Okuda^1^, Aoi Ikegami^1^, Asami Kitagawa^1^, Koudai Furuta^1^, Kana Izumi^1^, Eiko Shimizu^1^, Takashi Nishizono^1^, Makoto Fujiwara^1^, Tomohiro Miyasaka^8^, Shigeo Takamori^9^, Hiroshi Takayanagi^10^, Keizo Nishikawa^11^, Toshihiko Kobayashi^12^, Noriko Toyama-Sorimachi^12^, Makoto Yamashita^13^, Toshiya Senda^14^, Takatsugu Hirokawa^15,16^, Haruhiko Bito^17,18^ & Kiyotaka Nishikawa^1^*

*Corresponding author : Kiyotaka Nishikawa

**Email:** [knishika@mail.doshisha.ac.jp](mailto:knishika@mail.doshisha.ac.jp)

**This PDF file includes:**

Figures S1 to S13

Tables S1 to S3

SI References


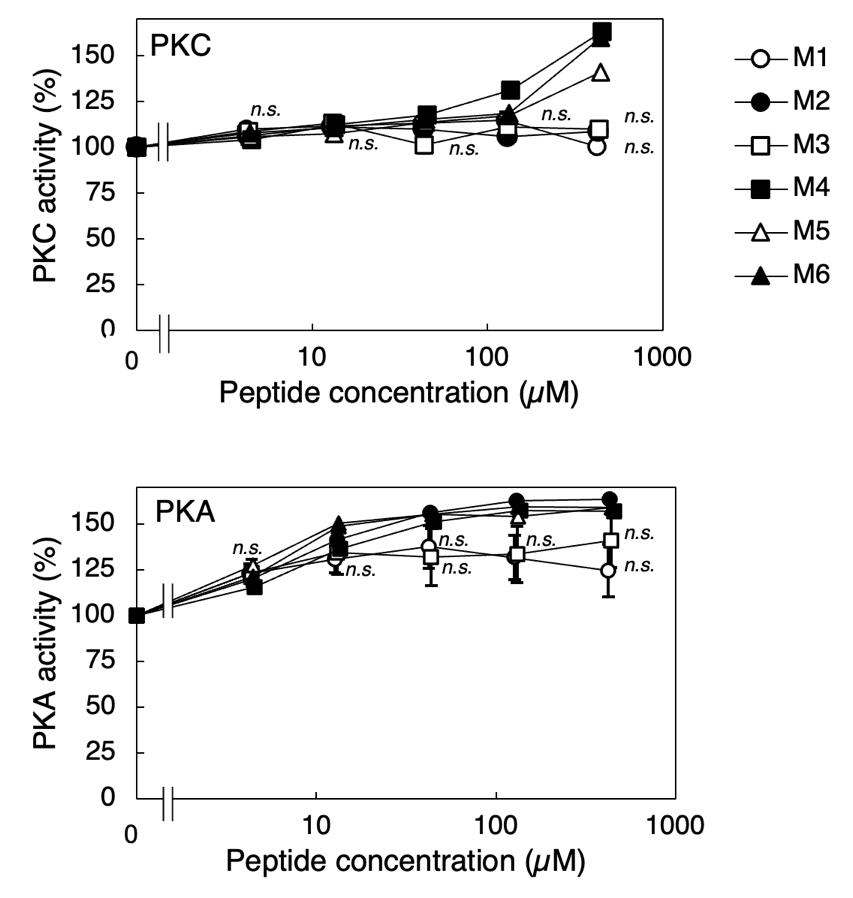


**Fig. S1. Monomer peptides (M1-M6) show no inhibitory effects on the kinase activity of PKC or PKA.**

Synthetic substrate peptide (Arg-Arg-Arg-Arg-Arg-Lys-Gly-Ser-Phe-Arg-Arg-Lys-Lys) was synthesized and was phosphorylated by PKC (α,β,γ) (Promega, WI, USA) in the presence of the indicated amount of inhibitory peptides in the kinase buffer (0.2 mM MgCl_2_ , 1 mM Tris-HCl (pH 7.4), 40 µM DTT, 20 μM ATP, 120 μg/ml phosphatidylserine, 100 μM diacylglycerol, 80 μM CaCl_2_, and 0.05 µCi [γ -^32^ P]ATP) for 5 min at 37°C (upper panel). Synthetic substrate peptide (Arg-Arg-Arg-Arg -Ser-Ile-Ile-Phe-Ile) was synthesized and was phosphorylated by PKA catalytic subunit (Promega) in the presence of the indicated amount of inhibitory peptides in the kinase buffer (2 mM MgCl_2_ , 10 mM Tris-HCl (pH 7.0), 0.2 mM DTT, 20 μM ATP, and 0.005 µCi [γ -^32^ P]ATP) for 5 min at 37°C (lower panel). Data are presented as a percentage of the control value without an inhibitory peptide (*n* = 2-4, mean ± SEM). *n.s.*, not significant (compared with the control by one-way ANOVA followed by one-sided Dunnett’s test).


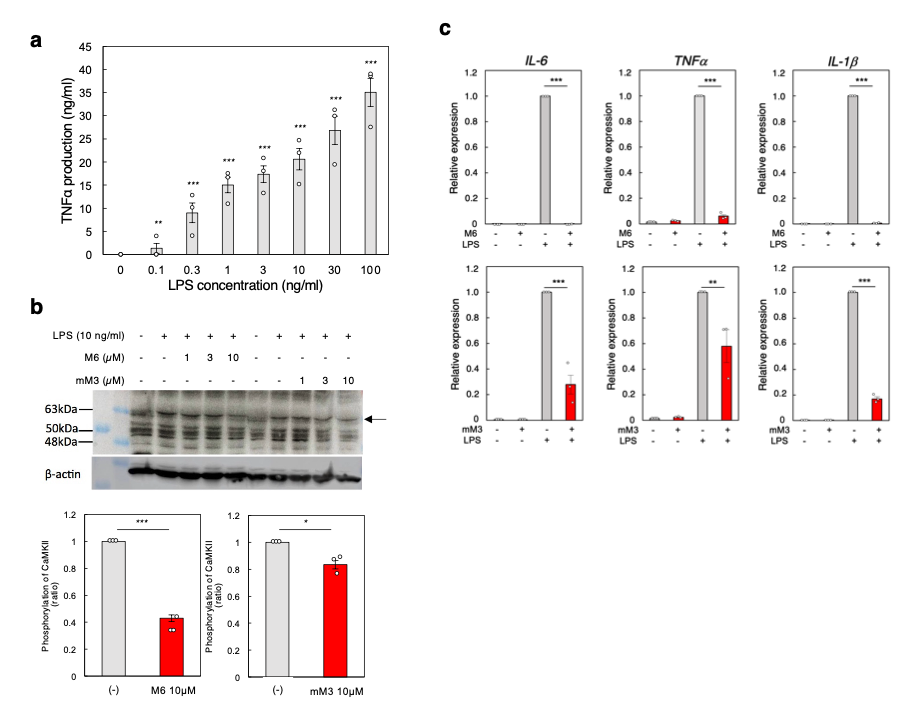


**Fig. S2.** **M6 and mM3 inhibit CaMKII activation and subsequent proinflammatory cytokine expressions in RAW264.7 cells.**

**a** LPS promotes tumor necrosis factor α (TNFα) production in RAW264.7 cells. RAW264.7 cells were treated with the indicated concentration of LPS for 24 h. The amount of TNFα produced in the culture medium was measured by ELISA. Data are presented as a percentage of the control value without peptide (*n* = 3, mean ± SEM). ***P* < 0.01; ****P* < 0.001 (compared with the control by ANOVA followed by one-sided Dunnett’s test). **b** The effects of M6 and mM3 on the activation of CaMKII in RAW264.7 cells. RAW264.7 cells were incubated with the indicated concentration of each peptide for 30 min, and then treated with 10 ng/ml LPS for 20 min. The cell lysates were analyzed by western blot using phosphorylated Ser286 specific antibody to detect the activation of CaMKII (upper panel). Data are representative of three independent experiments. The intensity of each band was quantitated and data are presented as the amount of phosphorylated CaMKII relative to that of β-actin (lower panel; *n* = 3, mean ± SEM). **P* < 0.05, ****P* < 0.001 (compared with no inhibitor by Student’s *t*-test). **c** The effects of M6 and mM3 on the expression of proinflammatory cytokine mRNAs in RAW264.7 cells. RAW264.7 cells were incubated with the indicated concentration of each peptide for 30 min, and then treated with10 ng/ml LPS for 3 h. The mRNA level of each proinflammatory cytokine was analyzed by quantitative RT-qPCR. Data are presented as the amount of each mRNA relative to that of LPS treatment only (*n* = 3, mean ± SEM). ***P* < 0.01, ****P* < 0.001 (compared with the control by ANOVA followed by one-sided Tukey’s test).


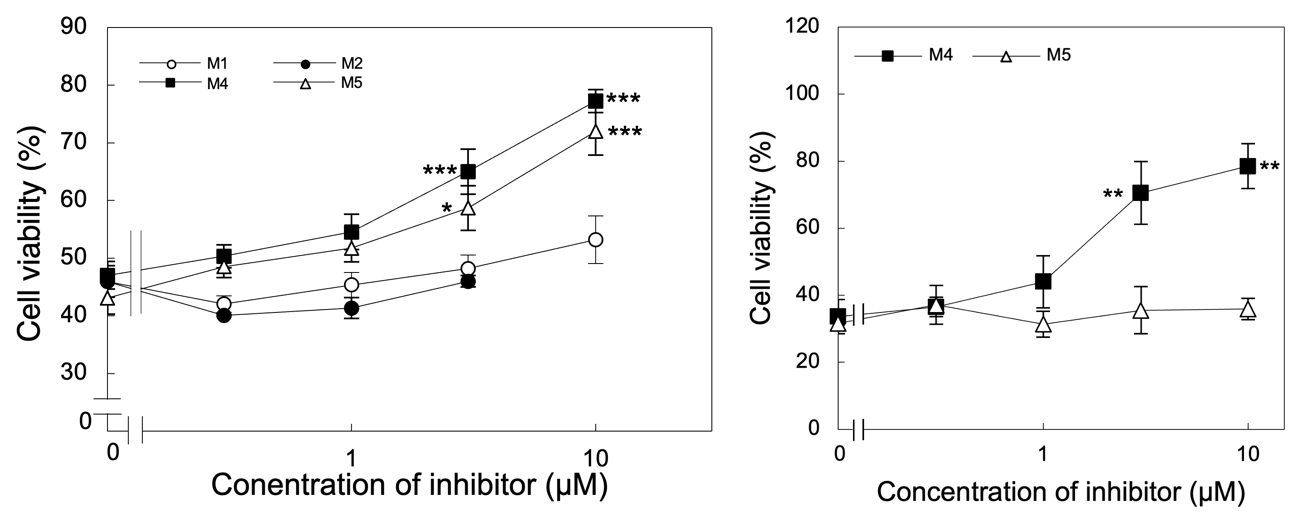


**Fig. S3. The effects of monovalent peptides on the cytopathicity induced by infection.**

MDCK cells were treated with peptides for 30 min and then infected with IAV strain PR8 at MOI = 20 (left panel; a single cycle infection) for 24 h or MOI = 0.001 (right panel; a multi-cycle infection) for 40 h. Data are presented as a percentage of the control value without infection (left panel; M1, M2: *n* = 3, M4: *n* = 11, M5: *n* = 7, virus alone: *n* = 16, right panel; *n* = 3, mean ± SEM). **P* < 0.05; ***P* < 0.01; ****P* < 0.001 (compared with the non-treated control cells by ANOVA followed by one-sided Dunnett’s test).

**Fig. S4. M3 has no direct effects on IAV particles.**

IAV strain PR8 particles (1×10^7^ pfu/ml) were incubated in the presence of M**
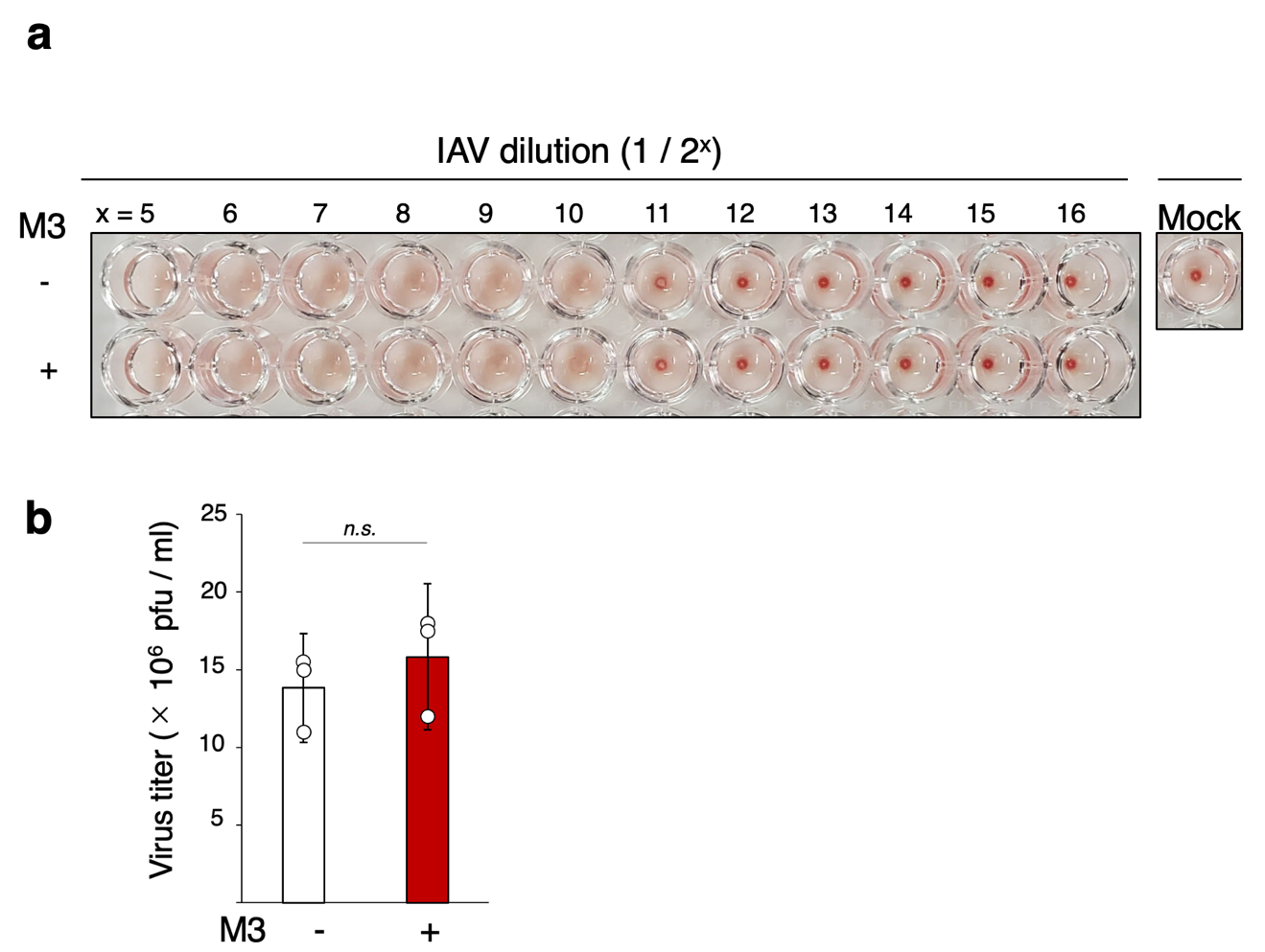
**3 (3.0 µM) or vehicle for 1 h at 37°C. The IAV particles were recovered by ultracentrifugation at 110,000 × g for 90 min at 4°C after loading on 30% sucrose PBS. **a** The effects of M3 on the IAV-induced hemagglutination. The obtained IAV particles were diluted as indicated, and then incubated with equal volumes of 0.75% chicken red blood cells for 1 h at 4°C in a U-bottom of 96-well plate. Hemagglutination was then visually analyzed (left panel). The right panel shows mock (no virus) control. **b** The effects of M3 on the virus titer. The obtained IAV particles were used for the determination of the virus titer using a regular plaque-forming assay using MDCK cell monolayers. *n.s.*, not significant (compared with vehicle by Student’s *t*-test).


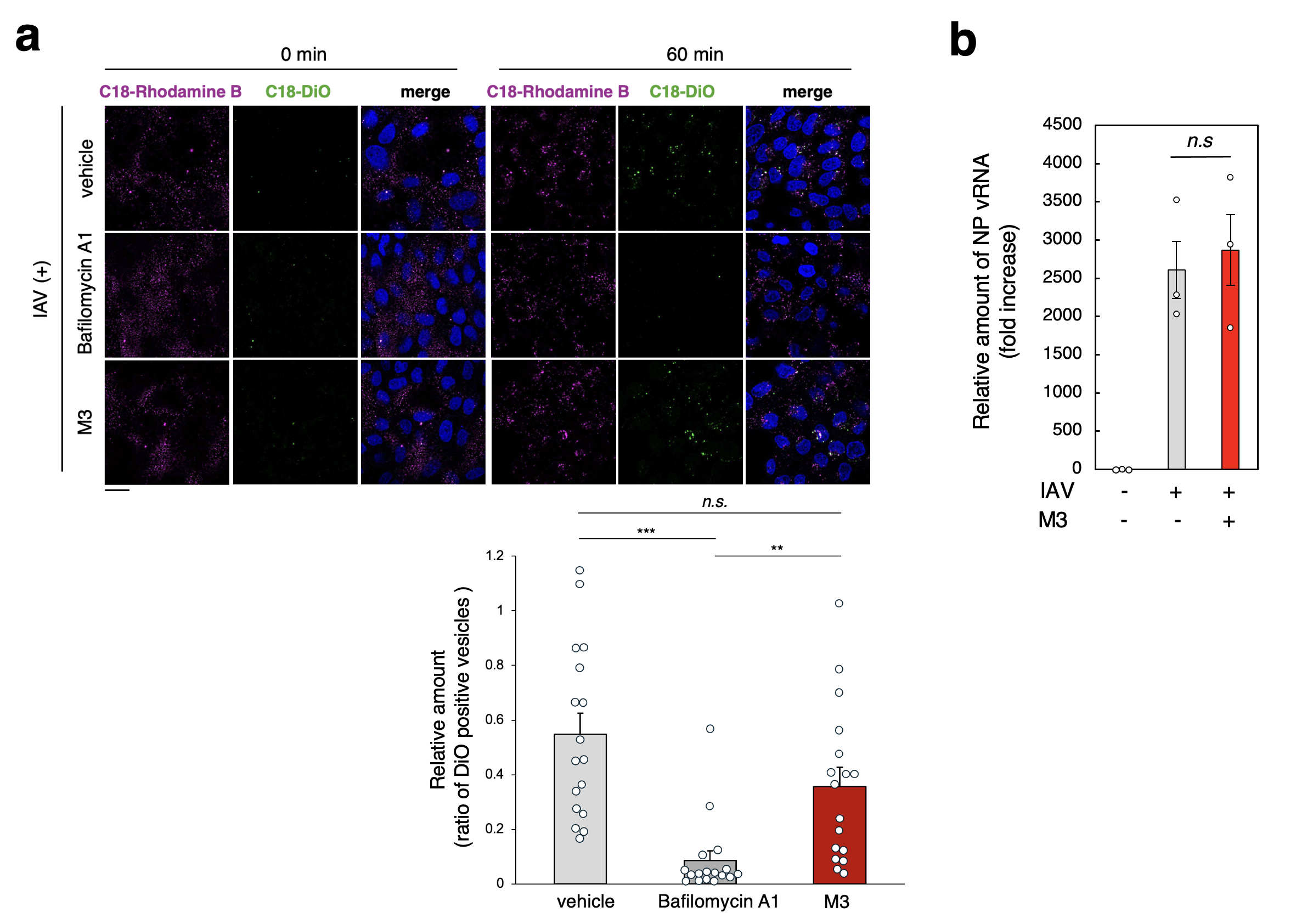


**Fig. S5. M3 does not affect the virus entry process into target cells.**

**a** To determine the effects of M3 on the fusion process after endocytosis of the virus, IAV strain PR8 was propagated in the presence of trypsin (1 µg/ml) for 72 h and then purified using chicken red blood cells (cRBC) as described previously (Protocol Exchange, DOI: 10.1038/protex.2014.027, 2014). Purified IAV particles (5 μg of total viral proteins) were labeled with two different lipophilic tracers, C18-Rhodamine B (red fluorescence) and C18-DiO (green fluorescence) (Molecular Probes), as described previously (J Virol., 80., 2013-2018, 2006). The IAV can be detected as a particle with red fluorescence only, because the fluorescence of DiO is quenched by that of Rhodamine B through the Förster resonance energy transfer. MDCK cells were infected with the labeled IAV at 30 MOI in the presence of M3 (3.0 µM), bafilomycin A1 (100 nM) (Cayman Chemicals), or vehicle for 30 min at 4°C. After washing (0 min, left panels), the cells were cultured at 37°C in the presence of M3 (3.0 µM), bafilomycin A1 (100 nM), or vehicle for 60 min. The nuclei were stained with 6.7 μg/ml Hoechst solution (DOJINDO, JAPAN). Fluorescent images were analyzed using laser scanning confocal microscopy. After fusion of the virus with the endosome membrane, these two tracers diffuse into the endosomal membrane, allowing the endosome vesicle to be detected by both red and green fluorescence as a result of DiO dequenching (right panels). Scale bar represents 10 μm. The ratio of DiO positive vesicles to C18-Rhodamine B positive vesicles was measured after a 60 min incubation using Image J software (lower panel; mean ± SEM, *n* [number of area with 15-25 nuclei] = 17, from 3 independent experiments). ***P* < 0.01, ****P* < 0.001 (by Tukey’s test). *n.s.*, not significant. M3 did not affect the fusion process, while bafilomycin A1, which inhibits the vacuole H^+^-ATPase and suppresses the fusion between the viral and cellular membranes after endocytosis of the virus, efficiently inhibited virus entry into cells.

**b** Quantification of NP vRNA level. MDCK cells were infected with IAV strain PR8 at 1 MOI in the presence of M3 (2.5 µM) or vehicle for 30 min at 4°C. After washing, the cells were cultured for 1 h at 37°C in the same condition without IAV. The relative amounts of NP vRNA were determined by quantitative RT-PCR using *GAPDH* as the reference gene. Data are presented as fold increase over the average RNA level at 1 h post infection without M3 (*n* = 3, mean ± SEM). *n.s.*, not significant (compared with vehicle by Student’s *t*-test).


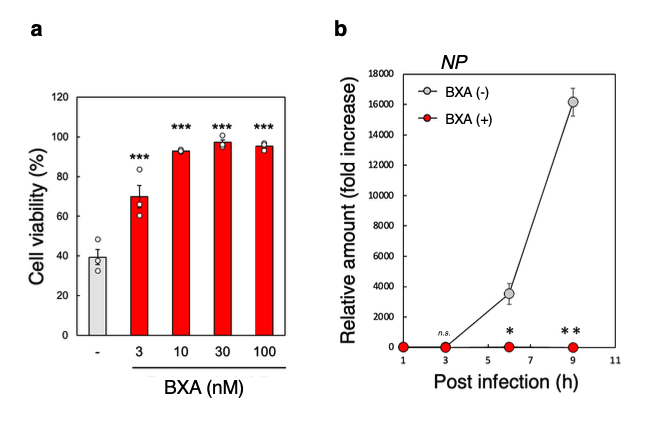


**Fig. S6. BXA shows marked anti-viral activity.**

**a** The effects of BXA on the cytopathicity induced by IAV infection. MDCK cells were incubated with the indicated concentrations of BXA for 30 min, and then infected with IAV strain PR8 at 10 MOI for 24 h. Data are presented as a percentage of the control value without infection (*n* = 3, mean ± SEM). ****P* < 0.001 (compared with the control by ANOVA followed by one-sided Dunnett’s test). **b** The effects of BXA on the expression of NP RNAs. MDCK cells were incubated with 100 nM BXA for 30 min, and then infected with IAV strain PR8 at 10 MOI for the indicated time periods. The relative amounts of NP total RNA were analyzed by quantitative RT-qPCR using *GAPDH* as the reference gene. Data are presented as fold increase over the average RNA level before infection (*n* = 3, mean ± SEM). **P* < 0.05; ***P* < 0.01 (compared with no BXA by Student’s *t*-test).


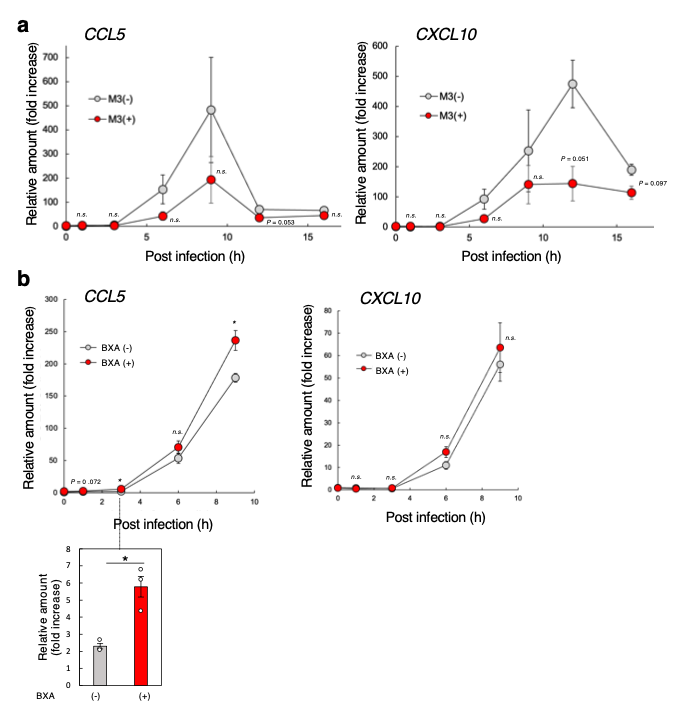


**Fig. S7. M3 specifically inhibits a cap-snatching event that functions at an early phase of infection.**

**a** The effects of M3 on the expression of CCL5 and CXCL10 mRNAs. MDCK cells were incubated with or without 3 μM M3 for 30 min, and then infected with IAV strain PR8 at 1 MOI for the indicated time periods. The relative amounts of each mRNA were analyzed by quantitative RT-PCR using *GAPDH* as the reference gene. Data are presented as fold increase over the average RNA level before infection (*n* = 3, mean ± SEM). *P* (compared with no inhibitor by Student’s *t*-test). **b** The effects of BXA on the expression of CCL5 and CXCL10 mRNAs. MDCK cells were incubated with or without 100 nM BXA for 30 min, and then infected with IAV strain PR8 at 1 MOI for 6 h. The relative amounts of each mRNA were analyzed by quantitative RT-PCR using *GAPDH* as the reference gene. Data are presented as fold increase over the average RNA level before infection (*n* = 3, mean ± SEM). **P* < 0.05 (compared with no inhibitor by Student’s *t*-test).


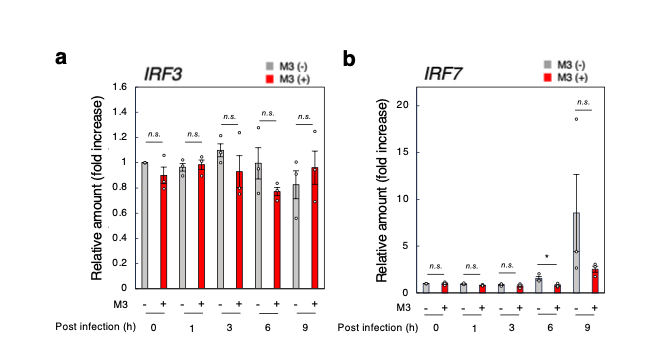


**Fig. S8. The effect of M3 on the expression of IRF3 and IRF7 mRNAs at an early stage of infection.**

**a, b** The effects of M3 on the expression of IRF3 (**a**) and IRF7 (**b**) mRNAs. MDCK cells were incubated with or without 3 μM M3 for 30 min, and then infected with IAV strain PR8 at 1 MOI for the indicated time periods. The relative amounts of each mRNA were analyzed by quantitative RT-PCR using *GAPDH* as the reference gene. Data are presented as fold increase over the average RNA level before infection (*n* = 3, mean ± SEM). **P* < 0.05; (compared with no M3 by Student’s *t*-test).


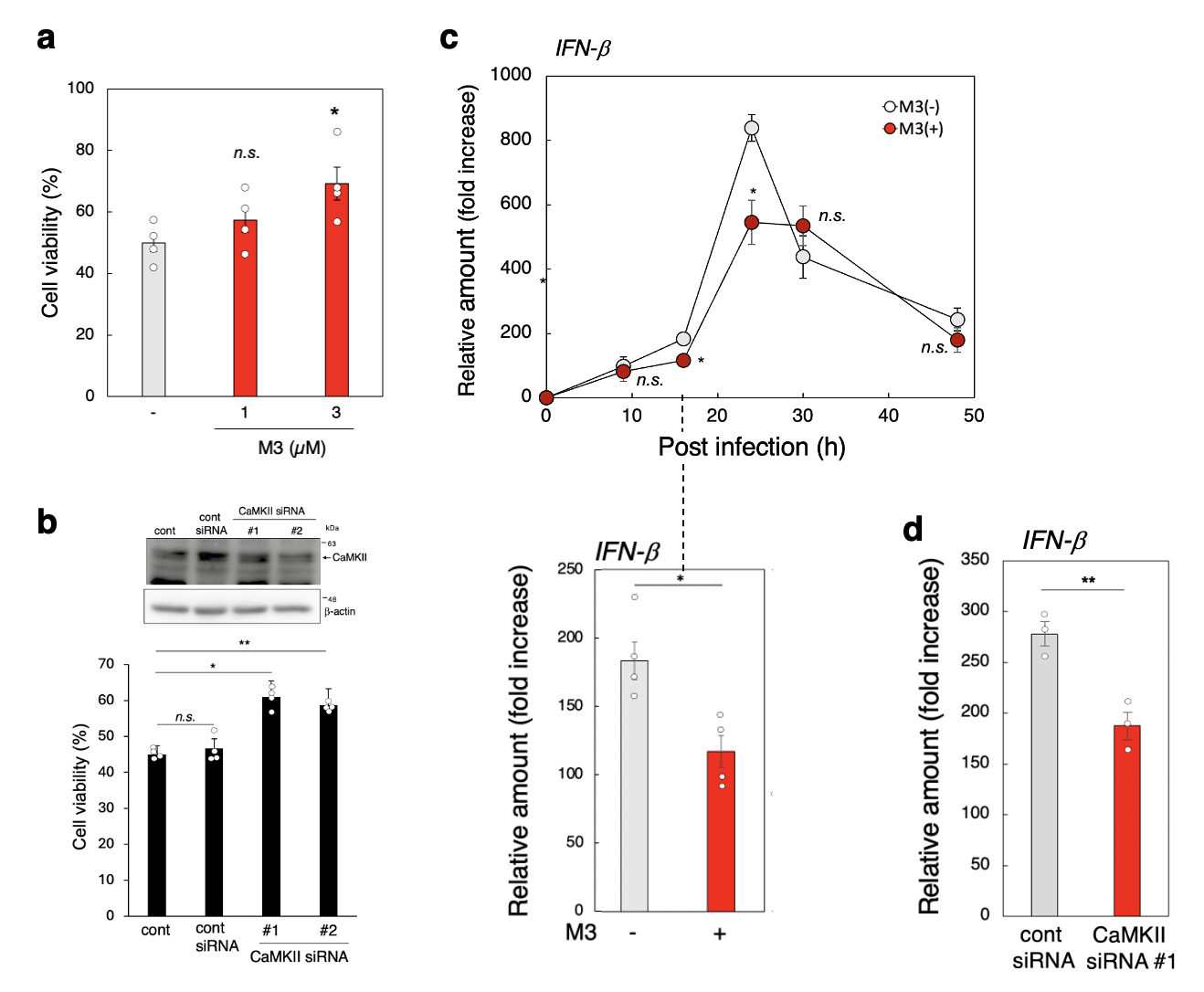


**Fig. S9.** **Antiviral activities of M3 in A549 cells.**

**a** The effects of M3 on the cytopathicity induced by IAV infection. A549 cells were incubated with the indicated concentrations of M3 for 30 min, and then infected with IAV strain PR8 at 10 MOI for 48 h. Data are presented as a percentage of the control value without infection (*n* = 4, mean ± SEM). **b** The effects of knock down of CaMKII on the cytopathicity. A549 cells were treated with 60 nM siRNA for CaMKII or control siRNA for 48 h and the lysates were analyzed using anti-CaMKII antibody (#4436, Cell Signaling Technology) (upper panel). Knock down of CaMKII was performed using 2 different siRNAs (Japan Bio Services, Japan). CaMKII target sequences were 5’-CACGACCATCCTGAACCCACA-3’ (#1) and 5’-CAGGATCTCTGACATCCTGAA-3’ (#2). The transfected cells were infected with IAV strain PR8 at 10 MOI for 48 h. Data are presented as a percentage of the control value without infection (*n* = 3 mean ± SEM). **P* < 0.05; ***P* < 0.01 (compared with control by ANOVA followed by one-sided Dunnett’s test). **c** The effects of M3 on the expression of IFNβ mRNA. A549 cells were incubated with or without 3 μM M3 for 30 min and then infected with IAV at 1 MOI for the indicated time. The relative amount of IFNβ mRNA was determined by RT-qPCR using *GAPDH* as the reference gene. Data for 9 h after infection are enlarged (lower panel). Forward and Reverse primers are as follows; 5’-GATGCTCCAGAACATCTTTGCTATT-3’ and 5’-GTTTTTCTTCCAGGACTGTCTTCAG-3’, respectively. Data are presented as the fold increase over the average RNA level before infection (*n* = 3 - 4, mean ± SEM). **P* < 0.05 (compared with no inhibitor by Student’s *t*-test). **d** The effects of knock down of CaMKII on the expression of IFNβ mRNA. A549 cells with knock down of CaMKII prepared as described above were infected with IAV at 1 MOI for 16 h. The relative amount of IFNβ mRNA was determined by RT-qPCR using *GAPDH* as the reference gene. Data are presented as the fold increase over the average RNA level before infection (*n* = 3, mean ± SEM). ***P* < 0.01 (compared with control by Student’s *t*-test).


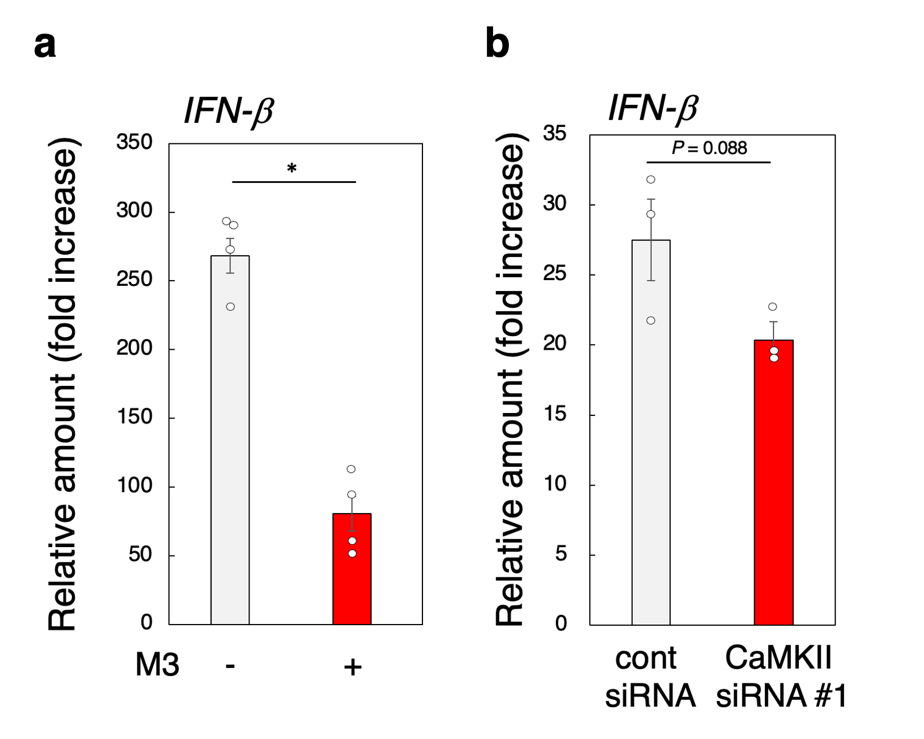


**Fig. S10.** **M3 inhibits the expression of IFNβ mRNA induced by a RIG-I ligand.**

**a** The effect of M3 on the expression of IFNβ mRNA induced by a RIG-I ligand. A549 cells were incubated with or without 2.5 μM M3 for 30 min, and then transfected with a RIG-I ligand, 5’ triphosphate hairpin RNA (3p-hpRNA; 25 ng/ml) (InvivoGen) for 16 h. The relative amount of IFNβ mRNA was determined by RT-qPCR using *GAPDH* as the reference gene. Data are presented as the fold increase over the average mRNA level in control cells (*n* = 4, mean ± SEM). **P* < 0.05 (compared with no inhibitor by Student’s *t*-test). **b** The effect of knock down of CaMKII on the expression of IFNβ mRNA induced by a RIG-I ligand. A549 cells with knock down of CaMKII prepared as described in Fig. S9b were transfected with 25 ng/ml 3p-hpRNA for 16 h. The relative amount of IFNβ mRNA was determined by RT-qPCR. Data are presented as the fold increase over the average mRNA level in control cells (*n* = 3, mean ± SEM). *P* = 0.088 (compared with no inhibitor by Student’s *t*-test).


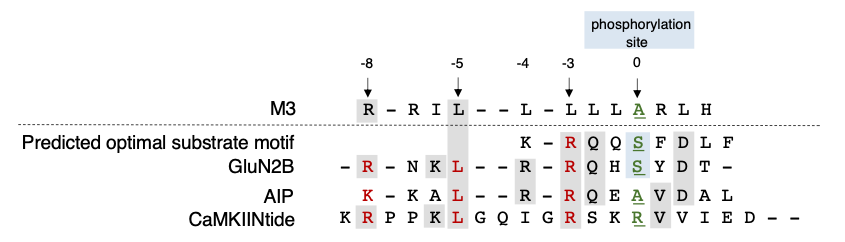


**Fig. S11.** **The sequence alignment of M3 and other substrate and inhibitory peptides.**

The sequences of indicated peptides are shown. Position 0 corresponds to the phosphorylation site of substrates, and is showed in green. Conserved amino acid at each position is highlighted in gray. Amino acids at positions -8, -5, and -3, which have been shown to play important roles in the binding to CaMKII-KD, are shown in red.


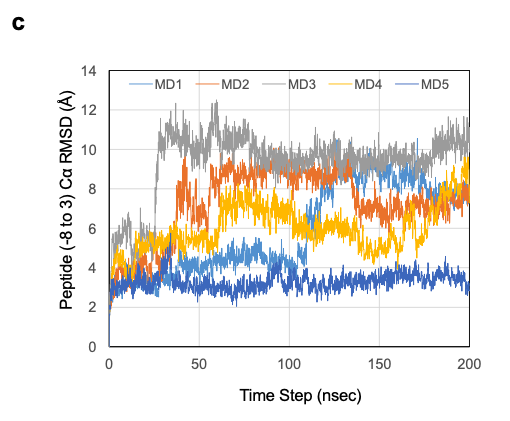

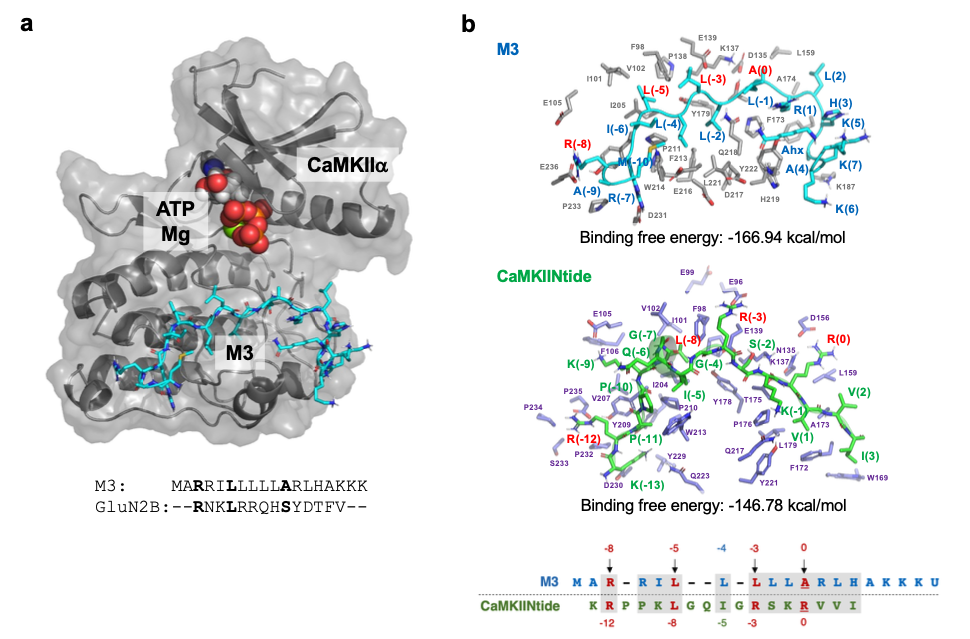
**Fig. S12.** **M3 binds to CaMKII-KD in a different manner from that of CaMKIINtide.**

Overview of the proposed CaMKII/M3 binding model (**a**) and closeup view of the binding sites with peptides for M3 and CaMKIINtide (**b**). The CaMKII/M3 binding model was predicted using crystal structure of CaMKII/GluN2B complex crystal (PDB-ID: 7UJP) as a template. The sequence alignment between M3 and GluN2B is shown in bottom (**a**). The complex modeling including CaMKII was constructed using Prime (1) (Schrödinger, LLC, NY, USA). We also conducted molecular dynamics (MD) simulations to assess the stability of the constructed binding model (**c**). Five independent MD simulations with different initial velocities for CaMKII/M3 complex model was performed using Desmond v2.3 (2) (Schrödinger, LLC, NY, USA). The OPLS3e force field was used for the simulations (3). The initial model structure was refined using the Protein Preparation wizard in Maestro (Schrödinger, LLC, NY, USA) and placed into TIP3P water molecules with periodic boundary conditions using an orthorhombic 10 Å layer simulation box. The system was neutralized, and an ionic force of 0.15 M was set by adding Na^+^ and Cl^−^ ions. After minimization and relaxation of the model, the MD production phase was performed for 200 ns with a time step of 2 fs in an isothermal–isobaric (NPT) ensemble at 300 K and 1 bar using a Nose–Hoover thermostat. The long-range electrostatic interactions were computed using the Smooth Particle Mesh Ewald method. The MD trajectories were saved every 10 ps for analysis. All system setups were performed using the Maestro software 2020-3 (Schrödinger, LLC, NY, USA). The obtained trajectory was processed utilizing the AMBER11 tools for the calculations of peptide RMSDs and representative structure generation. The final model was selected based on both the binding free energy and the stability of the peptide RMSD values. Binding free energy of peptide of the representative structure from last 100 ns of most stable production run of M3 complex and crystal structure of CaMKII/CaMKIINtide (PDB-ID: 3kl8) was calculated using MM-GBSA (Schrödinger, LLC). In this work, the model resulting from the MD5 calculation was selected as the final CaMKII/M3 binding model.


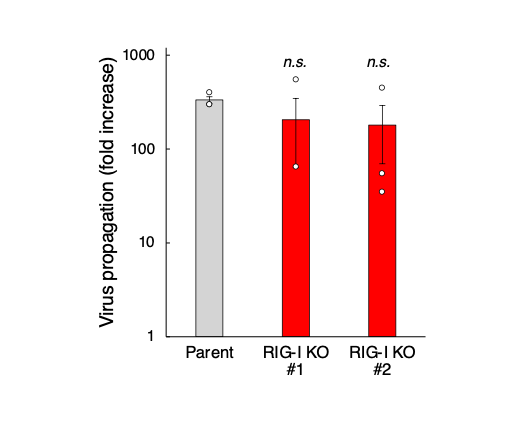


**Fig. S13. *RIG-I* knockout does not significantly affect the virus propagation.**

The effect of *RIG-I* knockout on virus propagation after IAV infection. MDCK-derived *RIG-I* knockout clones were infected with IAV strain PR8 at 0.2 MOI for 16 h. The virus titer in the supernatant was determined by a plaque assay. Data are presented as a fold increase over the initial virus titer (*n* = 3, mean ± SEM). *n.s.*, not significant

(compared with parental cells by ANOVA followed by one-sided Dunnett’s test).

**
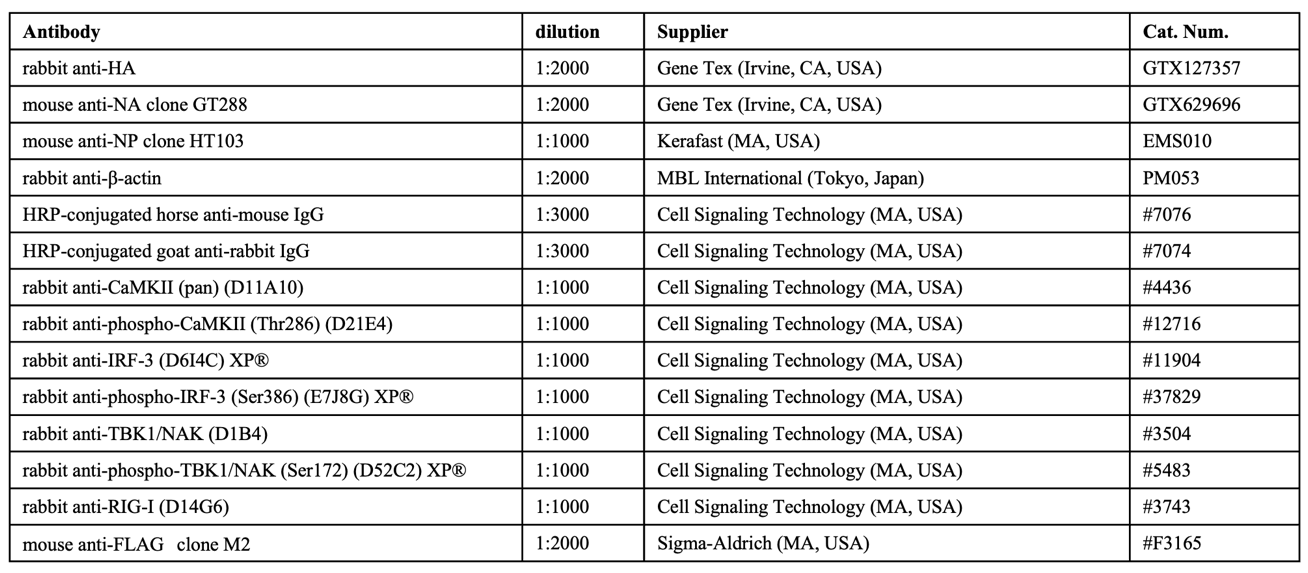
Table S1. Antibodies used in this study.**

**Table S2. Cell lines used in this study.**

**
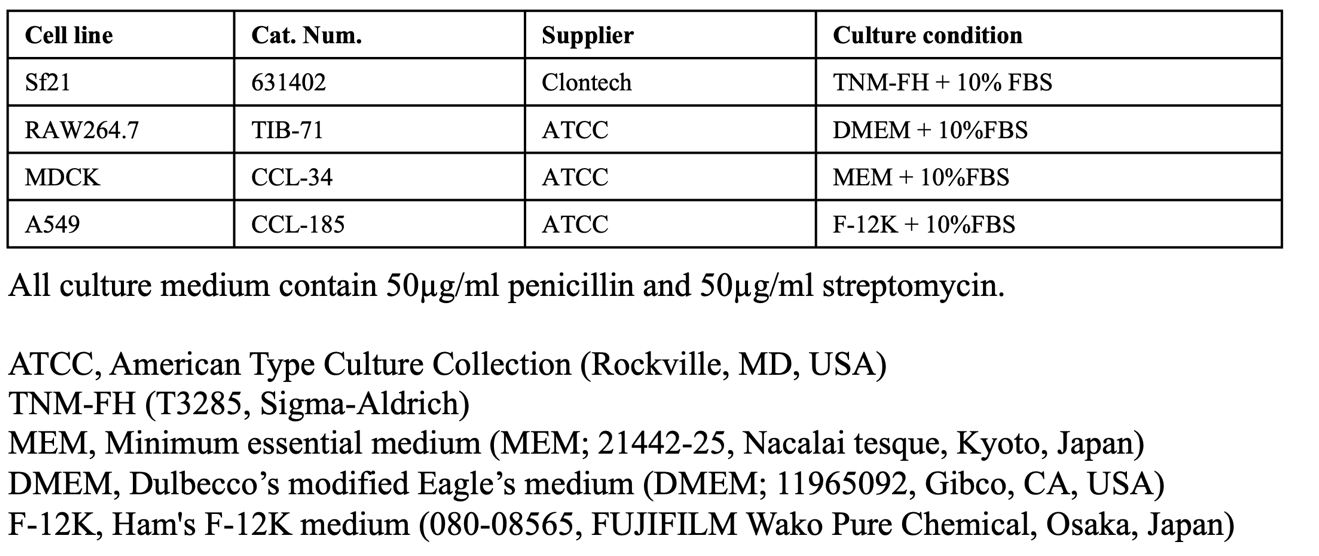
**

**
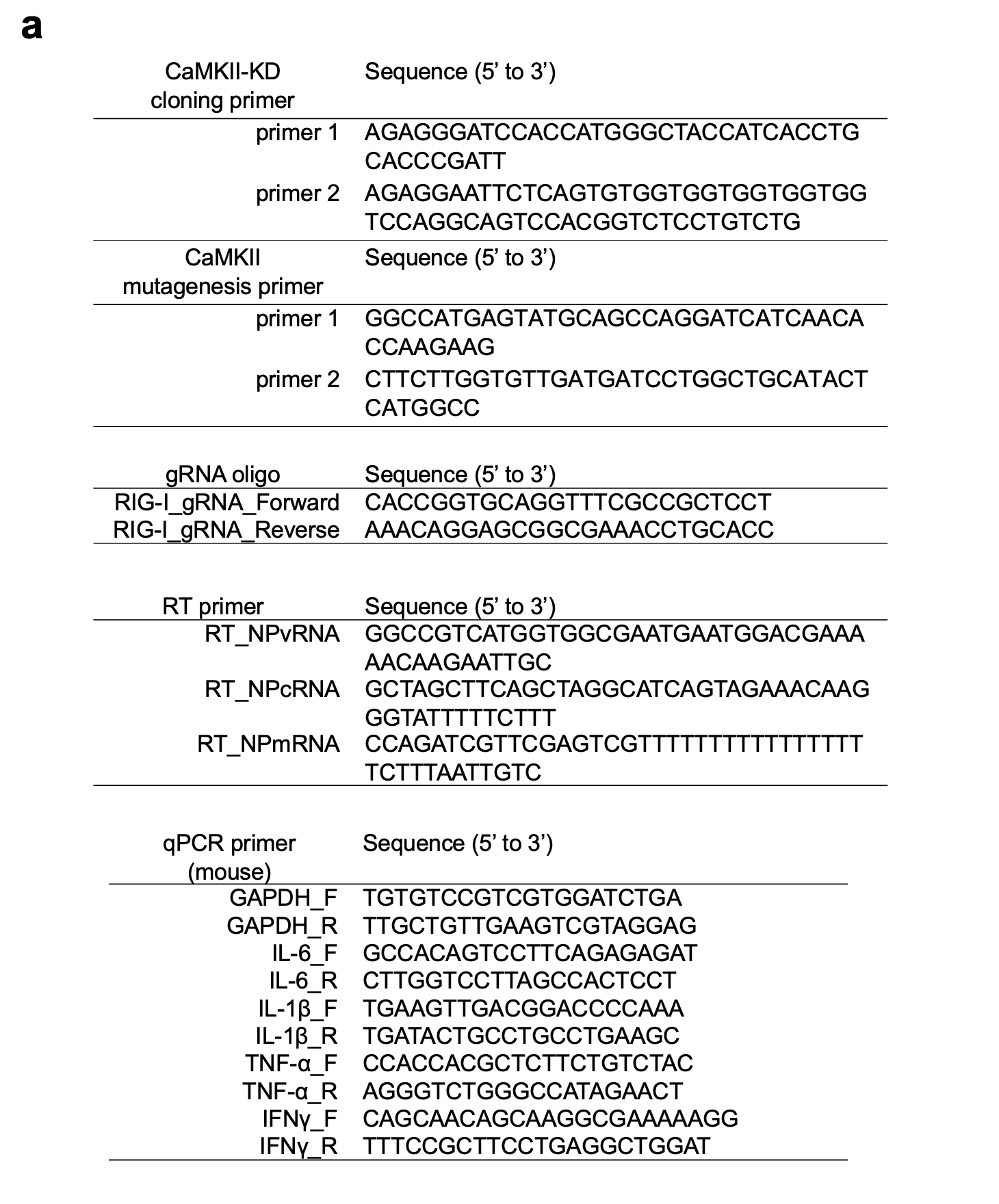
Table S3. Primers and Oligo DNAs used in this study.**


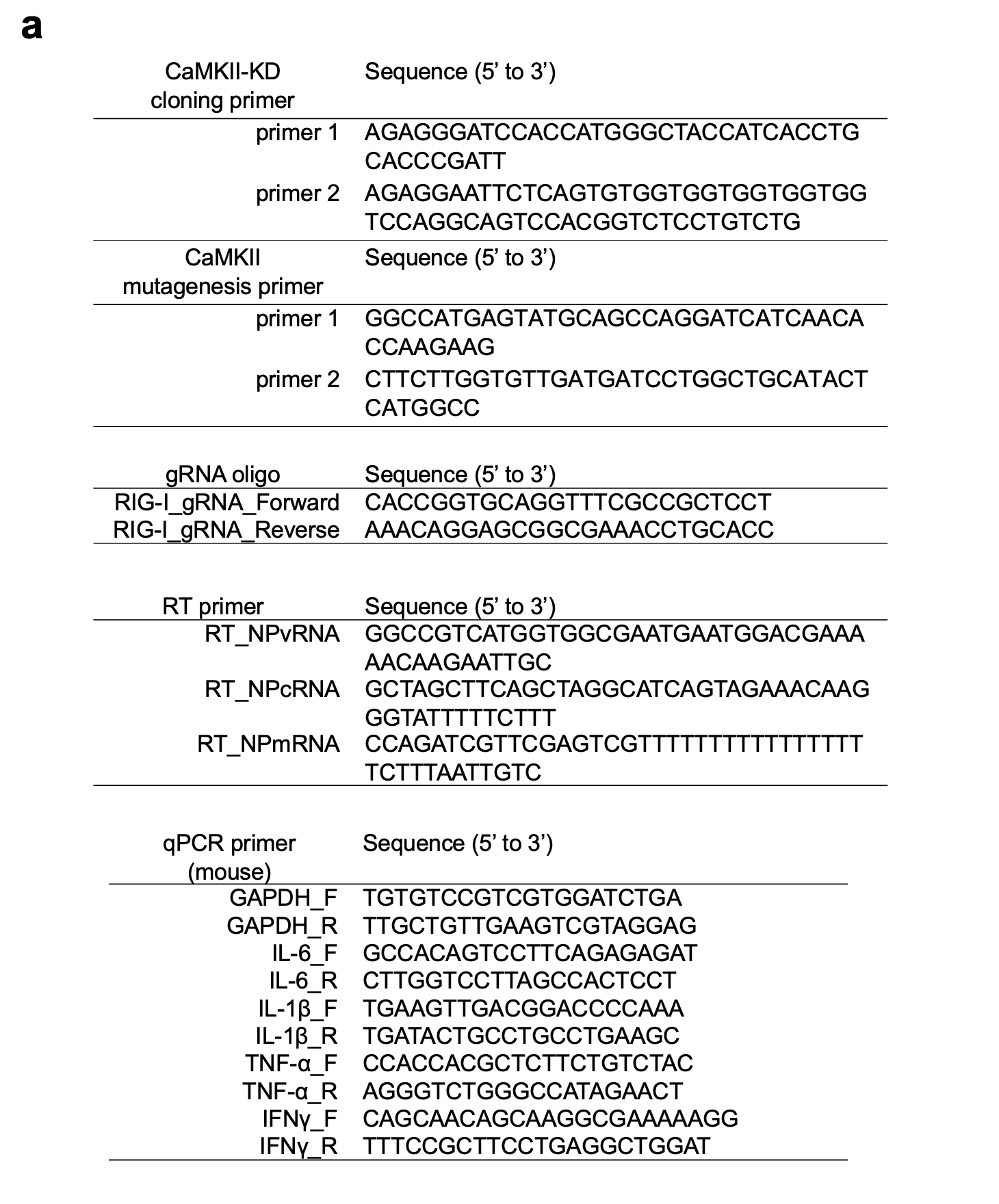


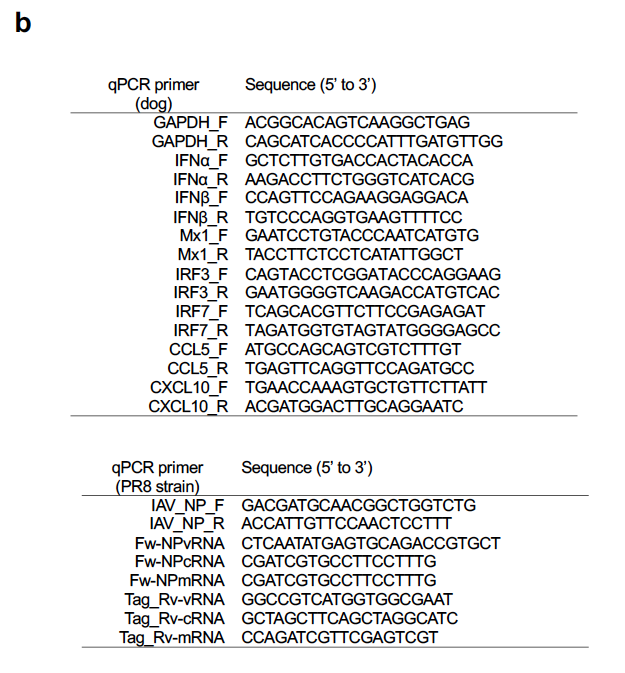

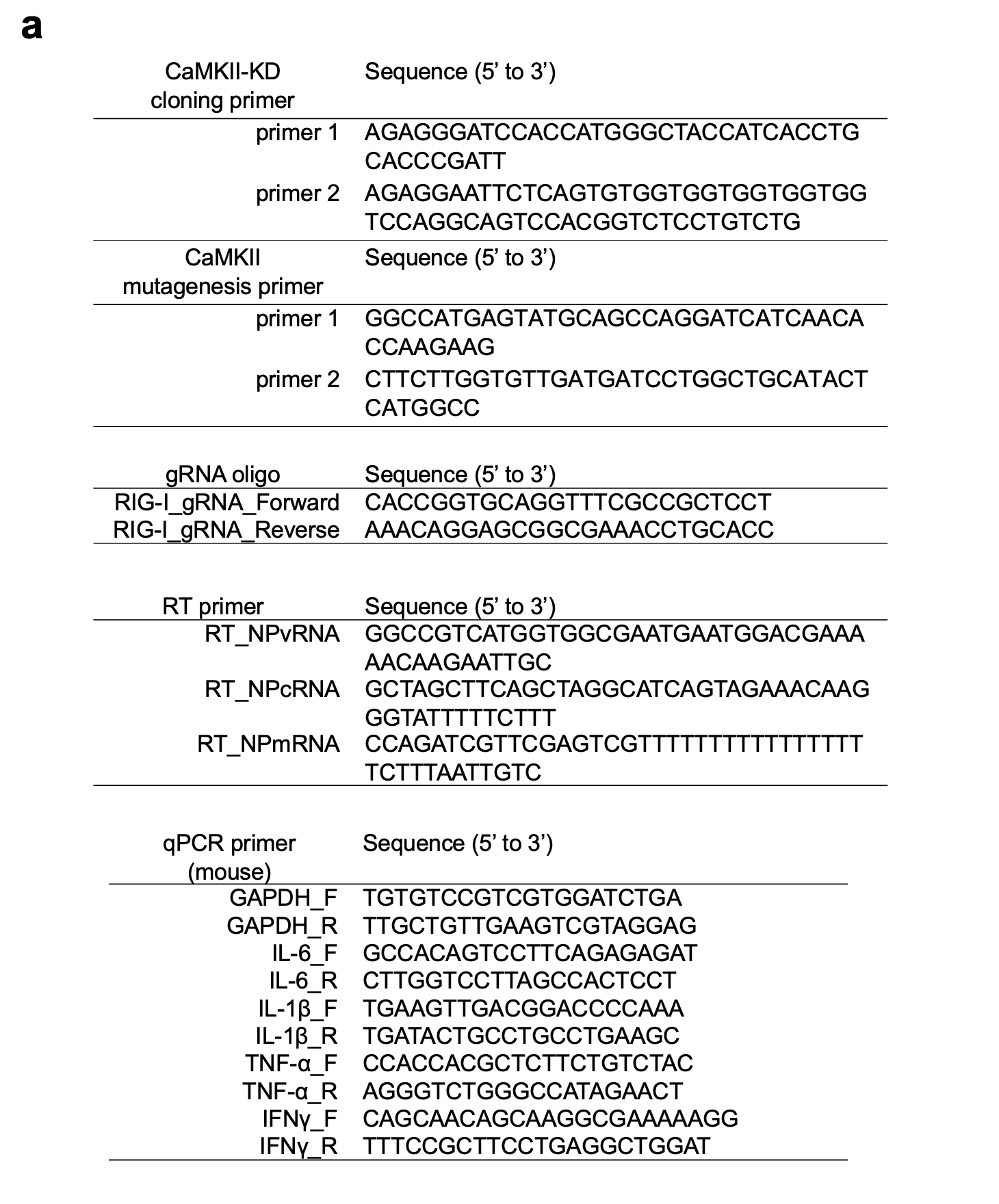


**SI References**

1. Jacobson, M. P., Pincus, D. L., Rapp, C. S., Day, T. J. F., Honig, B., Shaw, D. E., Friesner, R. A., A Hierarchical Approach to All-Atom Protein Loop Prediction, Proteins: Structure, Function and Bioinformatics, 55, 351-367 (2004).
2. Kevin J. Bowers, Edmond Chow, Huafeng Xu, Ron O. Dror, Michael P. Eastwood, Brent A. Gregersen, John L. Klepeis, Istvan Kolossvary, Mark A. Moraes, Federico D. Sacerdoti, John K. Salmon, Yibing Shan, and David E. Shaw, Scalable Algorithms for Molecular Dynamics Simulations on Commodity Clusters, Proceedings of the ACM/IEEE Conference on Supercomputing (SC06), Tampa, Florida, 2006, November 11-17
3. Harder, E. Damm, W., Maple, J.; Wu, C., Reboul, M., Xiang, J.Y., Wang, L., Lupyan, D., Dahlgren, M.K., Knight, J.L., Kaus, J.W., Cerutti, D.S., Krilov, G., Jorgensen, W.L., Abel, R., Friesner, R.A., OPLS3: A Force Field Providing Broad Coverage of Drug-like Small Molecules and Proteins, J. Chem. Theory Comput., 12,12(1), 281-96 (2016).
